# Identification and Validation of a Novel Prognosis Prediction Model in Adrenocortical Carcinoma by Integrative Bioinformatics Analysis, Statistics, and Machine Learning

**DOI:** 10.3389/fcell.2021.671359

**Published:** 2021-06-07

**Authors:** Xin Yan, Zi-Xin Guo, Dong-Hu Yu, Chen Chen, Xiao-Ping Liu, Zhi-Wei Yang, Tong-Zu Liu, Sheng Li

**Affiliations:** ^1^Department of Biological Repositories, Zhongnan Hospital, Wuhan University, Wuhan, China; ^2^Department of Urology, Zhongnan Hospital, Wuhan University, Wuhan, China; ^3^Human Genetics Resource Preservation Center of Hubei Province, Wuhan, China

**Keywords:** adrenocortical carcinoma, bioinformatics, machine learning, hub genes, prognosis prediction model

## Abstract

Adrenocortical carcinoma (ACC) is a rare malignancy with poor prognosis. Thus, we aimed to establish a potential gene model for prognosis prediction of patients with ACC. First, weighted gene co-expression network (WGCNA) was constructed to screen two key modules (blue: P = 5e-05, R^2 = 0.65; red: P = 4e-06, R^2 = −0.71). Second, 93 survival-associated genes were identified. Third, 11 potential prognosis models were constructed, and two models were further selected. Survival analysis, receiver operating characteristic curve (ROC), Cox regression analysis, and calibrate curve were performed to identify the best model with great prognostic value. Model 2 was further identified as the best model [training set: P < 0.0001; the area under curve (AUC) value was higher than in any other models showed]. We further explored the prognostic values of genes in the best model by analyzing their mutations and copy number variations (CNVs) and found that MKI67 altered the most (12%). CNVs of the 14 genes could significantly affect the relative mRNA expression levels and were associated with survival of ACC patients. Three independent analyses indicated that all the 14 genes were significantly associated with the prognosis of patients with ACC. Six hub genes were further analyzed by constructing a PPI network and validated by AUC and concordance index (C-index) calculation. In summary, we constructed and validated a prognostic multi-gene model and found six prognostic biomarkers, which may be useful for predicting the prognosis of ACC patients.

## Introduction

Adrenal cortical carcinoma (ACC) is a relatively rare malignant tumor (Lam, 2021). Forty percent of the cases had distant metastasis at the time of diagnosis (Ayala-Ramirez et al., 2013; Guillaume et al., 2014). Worst of all, most patients with ACC have to face poor prognosis. The morbidity age of ACC showed bimodal distribution in kids and in 40- to 50-year-old people (Rodriguez-Galindo et al., 2021). As for the prognosis of ACC patients, the 5-year survival rate for ACC patients was as reported as 50% according to recent data from the American Society of Clinical Oncology (ASCO). At present, many studies identified molecular biomarkers associated with ACCs to solve this problem (He et al., 2017). It means that predicting survival and prognosis of patients with the help of biomarkers has great application prospects. Thus, we aimed to construct a multi-gene signature and some prognostic biomarkers, which perhaps can help in predicting the prognosis of patients with ACC.

Weighted gene co-expression network (Langfelder and Horvath, 2008) has been widely used for potential biomarker selection (Lv et al., 2021). In the present study, by using GSE76019 [downloaded from Gene Expression Omnibus (GEO) database], a co-expression network was constructed to select two key modules related to survival state. Then we screened out hub genes in a co-expression network and further identified survival-associated genes among them by using GEPIA (Gene Expression Profiling Interactive Analysis) (Tang et al., 2017). Considering that Ridge, Elastic Net, and Lasso (least absolute shrinkage and selection operator) methods were widely used data mining methods especially for regression model construction (Ye et al., 2021), based on the survival-associated genes, all the three methods were included for constructing multiple-gene signatures by using the data from TCGA database. Two prognostic models were constructed, which could significantly predict the OS (overall survival) of patients with ACC. Moreover, we constructed two nomograms based on the two multiple-gene signatures, which were verified to predict the probability of the overall survival of patients with ACC well. The calibration curve indicated that nomogram of model 2 showed better prognostic value compared with model 1, which was regarded as the best prognostic model. Moreover, six genes were further screened out by protein–protein interaction (PPI) network construction, which were regarded as hub genes in this study. In conclusion, we constructed a multi-gene signature that might be an independent survival and prognostic biomarker for ACCs. The nomogram based on the best model for survival rate of ACC patient prediction might give more beneficial guidance for clinical practice, and six hub genes were also screened out, which might be novel prognostic biomarkers of patients with ACC.

## Materials and Methods

### Data Collection

Supplementary Figure 1 showed the flowchart for the present research. In total, four independent datasets with complete clinical information were retrieved from public databases in the present study. For the WGCNA analysis, dataset GSE76019 (Pinto et al., 2016) was retrieved from the GEO database^1^ to conduct WGCNA. GSE76019, performed on Affymetrix HT HG-U133 + PM array plate, included 34 ACCs. To search for the best regression model by using the Ridge (α = 0), Elastic Net (with α varying from 0.1 to 0.9), and Lasso (α = 1) methods, we downloaded the mRNA-seq data and clinical information of ACCs from The Cancer Genome Atlas (TCGA) database. After weeding out incomplete samples, 79 ACCs were included in this study. We randomly split them into two groups with a ratio of 2:1. The training set, including 52 samples, was selected to construct the regression models. Meanwhile, the rest of the 27 samples were used to preliminarily screen the best model and further validate our results (as an internal verification set). In order to conduct external validation for our results, two independent datasets were further retrieved. GSE19750 (Demeure et al., 2013) that performed on Affymetrix Human Genome U133 Plus 2.0 array included 44 ACCs and four normal adrenal glands. Dataset GSE76021, performed on Affymetrix Human Genome U133A Array, included 29 ACC samples. Supplementary Table 1 shows a detailed information including gender, age, stage, grade, and survival data of these datasets. The clinical characteristics of the training dataset and the internal validation dataset are shown in Supplementary Table 2. Further correlation analysis indicated that there was no significant difference between the two datasets (P of age: 0.870, P of gender: 0.660, P of stage: 0.051, and P of status: 0.816), which demonstrated that our grouping method was appropriate and reasonable.

To explore mutations and copy number variations (CNVs) of genes in the best model, we also downloaded the CNV data of the ACC samples from TCGA database.

### Preprocessing of Collected Data

For the microarray data of GSE19750, GSE76019, and GSE76021 from Affymetrix, we first downloaded the raw “CEL” data from the GEO database and further applied robust multiarray averaging (RMA) method for background adjustment and quantile normalization, by using the “affy” package in R software. For TCGA–ACC data, we first downloaded the count number expression profiles. The normalization and log2 transformation were next obtained via “DEseq.2” (Love et al., 2014) in R. Batch effects from non-biological technical biases were corrected using ComBat algorithm based on R package “sva” (Leek et al., 2012).

For WGCNA, 34 adrenocortical carcinoma samples from GSE76019 were included for WGCNA. Probe sets were filtered by their variances across all samples; only probes with variances ranked in the top 2,500 were selected for subsequent analyses. Microarray quality was assessed by sample clustering according to the distance between different samples in Pearson’s correlation matrices and average linkage. The expression values from TCGA–ACC data of the genes we identified in WGCNA were extracted to construct the regression models by using the Ridge, Elastic Net, and Lasso methods.

### Co-expression Network Constructing and Key Module Screening

First, gsg (goodSamplesGenes) and sample network methods were used to check the expression data profile of the top 2,500 genes from GSE76019. A standard of Z.Ku ≥ −2.5 was set to pick out qualified ACC samples for constructing a co-expression network. Based on “WGCNA” (Langfelder and Horvath, 2008) in R software, we constructed a co-expression network. We used three branch cutting methods used for classification of genes and construction of modules [manual (interactive) branch cutting approach, automatic single block analysis, and two block analysis]. Some essential parameters for branch splitting are shown here: minClusterSize = 30 and deepSplit = 2. Furthermore, a cutline for merging high-associated models was set through calculating the dissimilarity of module eigengenes (MEs). In order to identify key modules that correlated to survival state (the trait that interested us the most), we first calculated the gene significance (GS) with the aim of quantifying the correlation between genes and trait. Then we further defined the module significance (MS), which was the average GS of all the genes in a module. Based on the above analyses, the most positive correlation module and the most negative correlation module were regarded as key modules.

### Exploration of Potential Functions of Genes

GO enrichment (Ashburner et al., 2000) and KEGG pathway analyses (Kanehisa and Goto, 2000) were conducted *via* “clusterProfiler” (Yu et al., 2012) in R software. For the GO part, we only identified the biological process (BP). A gene set or KEGG signaling pathway with *P* < 0.05 was thought to be strong and enriched.

### Survival-Associated Gene Identification

In the present study, we chose genes that reached the standards of |cor.geneModuleMembership| > 0.8 and |cor.geneTraitSignificance| > 0.2 as hub genes. In order to find out genes associated with survival, we further performed overall survival (OS) analysis and diseases-free survival (DFS) analysis for these hub genes by using GEPIA (Tang et al., 2017). Genes were considered to be survival-associated genes when they showed significant *P*-value in both OS and DFS.

### Regression Model Construction and the Best Model Screening

Based on the training set, the expression values of survival-associated genes were extracted to construct models by using the Ridge, Elastic Net, and Lasso methods. Elastic Net regression utilizes a combination of Lasso (L1-norm) and Ridge (L2-norm) regularization penalties, and reduces overfitting by limiting coefficient magnitudes (Zou and Hastie, 2005). The regression equation can be seen in a previous study (Zou and Hastie, 2005). All of the three methods were performed by using R package “glmnet” (Friedman et al., 2010). A 10-fold cross-validation was performed for tuning parameter selection in the models. The 10-fold cross-validation was based on 1-SE (standard error) criteria. Samples in the training set were randomly divided into 10 parts, nine of which were trained in turn, while the remaining one was tested, and the average of the results of the 10 tests was used to estimate the accuracy of the algorithm. A total of 11 models would be constructed. We further got a gene signature from each model that contained the most useful prognostic biomarkers that the model was thought to have. To identify the best model among the 11 models, we calculated the risk score (RS) of every sample in the internal validation set based on these models. In each model, we calculated the RS by using the formula as follows:

$$\mathrm{Risk}\mathrm{score}=\sum_{i=1}^{n}Coef_{i}\times Exp_{i}$$

In which Coef represents the relative regression coefficient and Exp represents the expression value of each prognostic gene identified by the Ridge, Elastic Net, or Lasso methods. In the internal validation set, we used the risk score of each model to represent the model and further worked out the AUC to distinguish live ACCs from dead ACCs by performing receiver operating characteristic curve (ROC) analysis using the R package “plotROC” (Sachs, 2015). The model with the highest AUC was regarded as the best model, which was included for further analysis.

### Survival Analysis and Time-Dependent Receiver Operating Characteristic Analysis

We further worked out the risk score of each sample in all datasets based on the best model. ACCs in the training set, internal validation set, entire set, GSE19750, and GSE76021 were divided into high- and low-risk groups according to the median risk score we calculated. R package “survival” (Therneau, 2015) was used to perform survival analysis. In order to investigate the performance of the signature, R package “timeROC” (Blanche et al., 2013) was used to perform time-dependent ROC analysis. In this study, AUC values of 1, 3, and 5 years were explored, accurately, which could affect predictive accuracy.

### Cox Proportional Hazards Regression Analysis

To verify the prognostic value of the gene signature (best model), we included the risk score of the best model and some essential clinicopathological features (gender, age, tumor-, T-, N-, and M-stages, and Weiss score) for univariable Cox analysis of OS via TCGA-ACC dataset. Features with *P*-value < 0.05 were immediately chosen for multivariate Cox analysis. It would be clear if the gene signature was irrelevant to other clinical features for predicting OS of ACCs after Cox regression analysis. Visualization was applied by using “forestplot” (Aut and Aut, 2016) in R software.

### Gene Set Enrichment Analysis

In order to understand the lurking functions of the best multi-gene signature, we evaluated the median risk score based on GSE76019 (the dataset which was used for WGCNA analysis). After that, a total of 34 ACCs were split into two groups accurately (high- and low-risk groups). “c2.cp.kegg.v7.2.symbols.gmt,” “c5.all.v7.4.symbols.gmt,” and “c6.all.v7.4.symbols.gmt” were set as the reference gene sets, respectively. GSEA (Subramanian et al., 2005) was conducted between the two groups. In this study, KEGG signaling pathways reached the standards (nominal *P* < 0.05, | ES| > 0.6, gene size ≥ 100 and FDR < 25%) and were significantly enriched.

### Nomogram Construction and Validation

Cross-validation could deal with the overfitting of the model. Thus, we performed cross-validation before nomogram construction. Based on TCGA-ACC data, “rms” in R was used to construct the nomogram (Yizhou et al., 2013). Calibration curve was plotted to test the nomogram. The calibration curve was also used for visualization and the 45° line in the curve represents the best prediction. Besides, in order to evaluate the prediction effectiveness of the nomograms, the consistency index (C-index) (Michael and Ralph, 2010) between actual probability and predicted probability was measured. ROC curves were also plotted using R package “pROC” (Robin et al., 2011). In addition, in order to check the stability of the nomogram with and without ARG signature, we performed time-dependent (1-, 3-, and 5-year) ROC analysis. Beyond ROC, we also used R package “rmda” to perform decision curve analysis (DCA) (Vickers and Elkin, 2006) to make sure if the signature was of great value for predicting 1-, 3-, and 5-year survival probability by using TCGA–ACC data and GSE19750. Moreover, survival analysis was performed to explore the difference in survival between different nomogram point groups.

### Mutations and Copy Number Variations of Genes in the Best Model

To explore mutations and CNVs of genes in the best model, we included all the ACC samples with CNV data from TCGA database for this analysis. A webtool named CBio Cancer Genomics Portal^2^ was chosen for exploration of genetic alterations. Furthermore, we identified the association between genetic alterations and the clinical features of patients with ACC. We used chi-square or Mann–Whitney *U*-test to analyze the statistical significance of the result. By using the relative mRNA expression levels of genes in the best model, we also explored the relationship between CNVs and mRNA expression levels of these genes. ANOVA or Kruslal–Wallis test was chosen to test the result. Unpaired t-test was used to test the expression differences between shallow deletion and gain groups. Moreover, the association between CNVs of genes in the best model and survival of patients with ACC was also identified by performing survival analysis based on R package “survival.”

### Linear Discriminant Analysis, K-Nearest Neighbor, and Support Vector Machine

To verify the prognostic value of genes in the best model, we regarded genes as variables and relative mRNA expression values as variable values. Then we performed LDA, KNN, and SVM analyses. We used package “MASS” (Venables and Ripley, 2002) in R software for LDA. For KNN algorithm, we chose the best K parameter by adopting cross validation method based on R package “caret” (Kuhn, 2015). Then we performed KNN algorithm by using R packages “class” (Venables and Ripley, 2002) and “kknn” (Hechenbichler and Schliep, 2004). For SVM analysis, four kinds of algorithms were used in the present study based on “e1071” (Meyer, 2015) in R software [linear-SVM, polynomial-SVM, radial basis function (RBF) SVM, and sigmoid-kernel SVM]. R package “kernlab” (Karatzoglou et al., 2004) was used to assist with SVM feature selection. We used the data from TCGA as training set to first construct classifier in each analysis and further verify the value of the classifier by calculating the classification rate based on TCGA–ACC data, GSE19750, GSE76019, and GSE76021. In this study, we regarded genes in the best model as meaningful prognostic biomarkers when the accuracy of classification ≥ 0.50.

### Protein–Protein Interaction Network Construction to Screen Hub Genes in the Best Model

To find out some relative genes in the best model, we first constructed a PPI network of genes formed from this model by using the STRING (Szklarczyk et al., 2019). Then the PPI network was imported into the Cytoscape software^3^. We further calculated the degree of connectivity of each gene. In this study, genes with the highest degree were regarded as hub genes. We also analyzed and visualized the correlation among genes in the best model by performing Spearman analysis using R package “corrplot” (Wei and Simko, 2013) and “PerformanceAnalytics” (Peterson et al., 2014). We further performed ROC and calculated AUC and C-index to validate these hub genes.

## Results

### Constructing a Co-expression Network to Identify Key Modules

**FIGURE 1:**
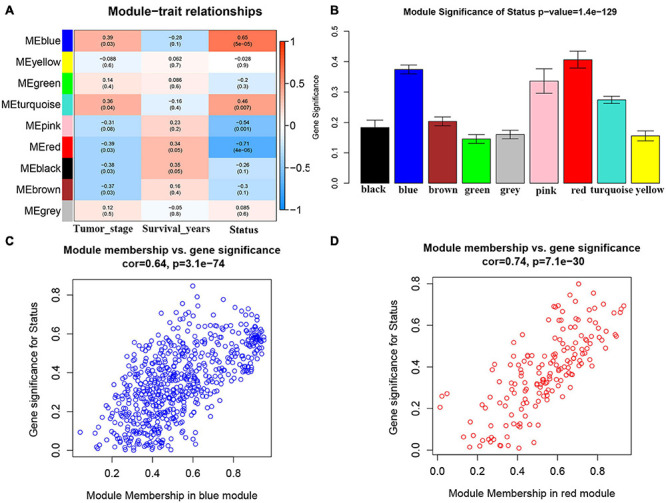
Identification of modules associated with the clinical traits of adrenocortical carcinoma (ACC). **(A)** Heatmap of the correlation between module eigengenes and clinical traits of ACC. **(B)** Distribution of average gene significance and errors in the modules associated with survival status of ACC. **(C)** Scatter plot of module eigengenes related to survival status in the blue module. **(D)** Scatter plot of module eigengenes related to survival status in the red module.

We first included 33 ACC samples to perform WGCNA analysis after weeding out the outlier samples (Supplementary Figure 2). According to the result, the beta (β) = 5 (scale free R2 = 0.89) was further set as the soft-thresholding value for further adjacency calculation (Supplementary Figure 3). Eventually, as shown in Supplementary Figure 4, in total, eight modules were identified. Genes not included in any other significant modules were included in the gray module, which were removed from subsequent analysis. Among the eight modules, the blue module was positively associated with survival status the most (*P* = 5e−05, R2 = 0.65). Meanwhile, the red module was the most negatively associated module (*P* = 4e−06, R2 = −0.71) (Figure 1A). As shown in Figure 1B, the MS of the two modules were also higher than the MS of any other module. The relationships between MM and GS in the blue module (cor = 0.64) and red module (cor = 0.74) were also significant as suggested by Figures 1C,D. Thus, we considered blue and red modules to be the key modules in this study. As shown in Supplementary Figures 5A,B, we also created a network heatmap and a classical MDS plot.

### Genes in Key Module-Associated Biological Pathways

According to GO biological process analysis, genes in the red module were enriched in four BPs including leukocyte migration, positive regulation of cell migration, regulation of G-protein-coupled receptor protein signaling pathway, and chemokine-mediated signaling pathway (Supplementary Figure 6A). As for genes in the blue module, in total, they were significantly correlated with 197 BPs (Supplementary Table 3). The top 10 enrichment BPs were organelle fission, nuclear division, chromosome segregation, nuclear chromosome segregation, mitotic nuclear division, sister chromatid segregation, mitotic sister chromatid segregation, microtubule cytoskeleton organization involved in mitosis, regulation of chromosome segregation, and mitotic spindle organization (Supplementary Figure 6B). When talking about KEGG pathway analysis, genes in the blue module were enriched in only three KEGG pathways including cell cycle, oocyte meiosis, and p53 signaling pathway (Supplementary Figure 6C).

### Survival-Associated Gene Identification

With the cutoff criterion (| cor.geneModuleMembership| > 0.8 and | cor.geneTraitSignificance| > 0.2), 107 genes (12 genes in the red module and 95 genes in the blue module) were identified in the co-expression network in total. Then we performed OS and DFS analysis for these 107 genes by using GEPIA. According to the results, high expression levels of 95 genes were obviously associated with poor OS for ACCs. Meanwhile, 100 genes showed significant *P*-values in DFS analysis. Ninety-three genes were common among them, which were regarded as survival-associated genes (Supplementary Table 4).

### Establishment of Two Multi-Gene Signatures for Predicting Overall Survival

We identified 93 biomarkers significantly related to survival of ACCs by using WGCNA analysis. With the aim of setting up a multiple-gene signature for prognosis in ACC patient prediction, we calculated relative regression coefficient of each gene using the Ridge, Elastic Net, and Lasso methods. The most powerful prognostic markers of the 93 biomarkers were screened out in each model as shown in Supplementary Figure 7. After that, 11 prognostic models were constructed, and the relative regression coefficients of the most powerful prognostic markers are shown in Supplementary Table 5. Combining the relative expression levels of the mRNA in the classifier and corresponding Lasso coefficients, we first worked out the risk scores of samples in the internal verification set. The distribution of risk scores of Ridge (Supplementary Figure 8A), Elastic Net with α varying from 0.1 to 0.9 (Supplementary Figures 8E–J), and Lasso model (Supplementary Figure 8K) of ACC patients based on TCGA–ACC data are shown. In all the models, it was obvious that the number of patients who died in the high-risk group was high, when compared with the low-risk group (Supplementary Figure 8). Furthermore, we worked out the AUC based on the risk score (Table 1). Two models, namely, model 1 (AUC = 0.676; α = 0.1) and model 2 (AUC = 0.676; α = 0.6) were finally identified, which were considered as the best candidate models.

**TABLE 1:** Area under the curve (AUC) of risk scores calculated by the Ridge (α = 0), Elastic Net (with α varying from 0.1 to 0.9), and least absolute shrinkage and selection operator (Lasso; α = 1) methods by using internal validation set.

**Models**	**Alive vs Dead**	
	**AUC**	**95% CI**
Ridge (α = 0)	0.670	0.445-0.896
Elastic Net (α = 0.1)	0.676	0.447-0.905
Elastic Net (α = 0.2)	0.670	0.440-0.901
Elastic Net (α = 0.3)	0.636	0.404-0.869
Elastic Net (α = 0.4)	0.574	0.347-0.801
Elastic Net (α = 0.5)	0.574	0.349-0.798
Elastic Net (α = 0.6)	0.676	0.457-0.895
Elastic Net (α = 0.7)	0.563	0.333-0.792
Elastic Net (α = 0.8)	0.551	0.323-0.780
Elastic Net (α = 0.9)	0.551	0.326-0.776
Lasso (α = 1)	0.534	0.308-0.760

**FIGURE 2:**
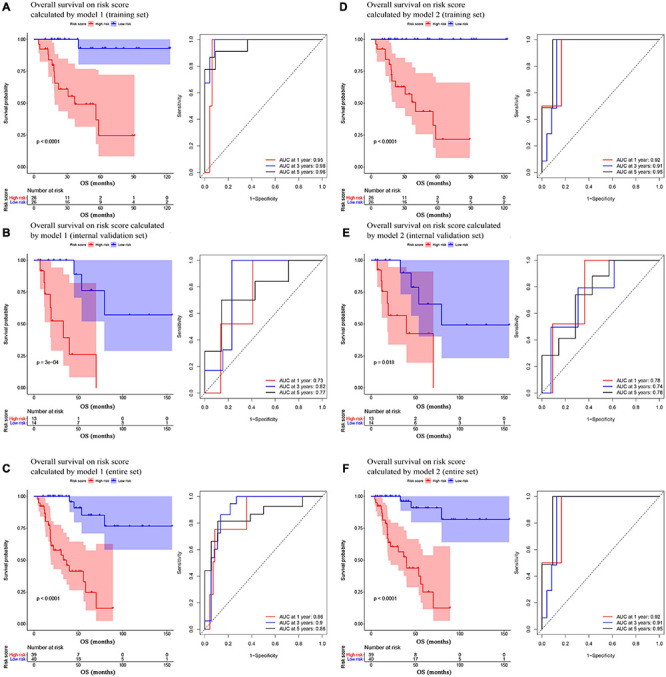
Survival analysis of the association between risk score calculated by model 1 and overall survival time in ACC, and time-dependent receiver operating characterictic (ROC) analyses at 1, 3, and 5 years in panels **(A)** training set, **(B)** internal validation set, and **(C)** entire set. Survival analysis of the association between risk score calculated by model 2 and overall survival time in ACC, and time dependent ROC analyses at 1, 3, and 5 years in panels **(D)** training set, **(E)** internal validation set, and **(F)** entire set.

In model 1 and model 2, ACCs in the training set were further split into two groups (low- and high-risk) via a median-risk score (5.0406 in model 1 and 16.5329 in model 2). In both models, ACC patients in the high-risk group occupied worse OS (Model 1: *P* < 0.0001, Figure 2A; model 2: *P* < 0.0001, Figure 2D). We also performed time-dependent ROC analysis. The results suggested that the AUC values of model 1 in the training set were 0.95 at 1 year, 0.98 at 3 years, and 0.96 at 5 years (Figure 2A). Meanwhile the AUC values of model 2 in the training set were 0.92 at 1 year, 0.91 at 3 years, and 0.95 at 5 years (Figure 2D).

### Validation of Model 1 and Model 2

**FIGURE 3:**
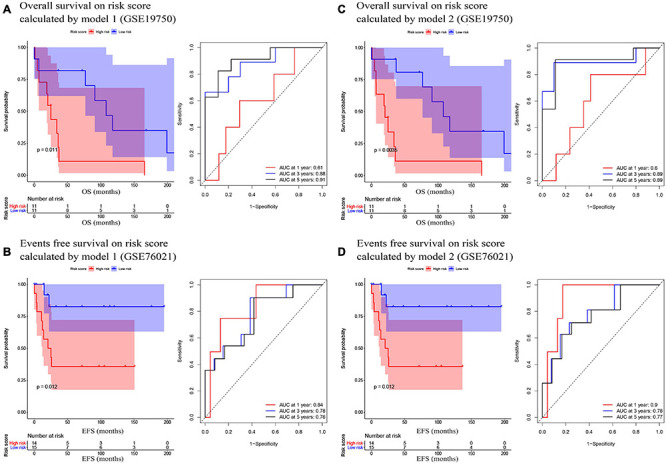
**(A)** Survival analysis of the association between risk score calculated by model 1 and overall survival time in ACC, and time dependent ROC analyses at 1, 3, and 5 years in GSE19750; **(B)** Survival analysis of the association between risk score by model 1 and events-free survival time in ACC, and time dependent ROC analyses at 1, 3, and 5 years in GSE76021; **(C)** Survival analysis of the association between risk score calculated by model 2 and overall survival time in ACC, and time dependent ROC analyses at 1, 3, and 5 years in GSE19750; **(D)** Survival analysis of the association between risk score by model 2 and event-free survival time in ACC, and time dependent ROC analyses at 1, 3, and 5 years in GSE76021.

In the present study, we used three validation sets (intervalidation set, GSE19750, and GSE76021) to validate the results we got from the training set. With the same method we mentioned in the training set, we calculated the risk score for each sample in these three validation sets based on model 1 and model 2. Samples in internal validation set, GSE19750, and GSE76021 were split into two groups relying on the median-risk score in each set. In model 1, according to the survival analysis, the high-risk group had lower survival rate compared with the low-risk group in all the validation sets (internal validation set: *P* = 3E−04; GSE19750: *P* = 0.011; GSE76021: *P* = 0.012) as the training set suggested (Figures 2B, 3A,B). The same conclusion was reached in the entire TCGA-ACC dataset (Figure 2C). As for the results of time-dependent ROC analysis, the prognostic accuracy of model 1 in the internal validation set was 0.73 at 1 year, 0.82 at 3 years, and 0.77 at 5 years (Figure 2B); the prognostic accuracy of model 1 in the entire TCGA-ACC dataset was 0.86 at 1 year, 0.90 at 3 years, and 0.96 at 5 years (Figure 2C); the prognostic accuracy of model 1 in GSE19750 was 0.61 at 1 year, 0.88 at 3 years, and 0.91 at 5 years (Figure 3A); the prognostic accuracy of model 1 in GSE76021 was 0.84 at 1 year, 0.78 at 3 years, and 0.76 at 5 years (Figure 3B). As for model 2, the high-risk group was associated with poorer survival compared with the low-risk group (internal validation set: *P* = 0.018; entire TCGA-ACC dataset: *P* < 0.0001; GSE19750: *P* = 0.0035; GSE76021: *P* = 0.012; Figures 2E,F, 3C,D). Moreover, the prognostic accuracy of model 2 in the internal validation set was 0.78 at 1 year, 0.74 at 3 years, and 0.76 at 5 years (Figure 2E); the prognostic accuracy of model 2 in the entire TCGA-ACC dataset was 0.92 at 1 year, 0.91 at 3 years, and 0.95 at 5 years (Figure 2F); the prognostic accuracy of model 2 in GSE19750 was 0.60 at 1 year, 0.89 at 3 years, and 0.89 at 5 years (Figure 3C); the prognostic accuracy of model 2 in GSE76021 was 0.90 at 1 year, 0.78 at 3 years, and 0.77 at 5 years (Figure 3D). Both the two models showed great prognostic values of patients with ACC.

### Prognostic Value of Model 1 and Model 2

**FIGURE 4:**
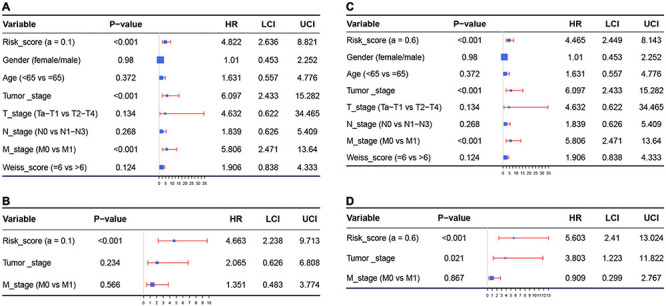
**(A)** Forest plot summary of analyses of overall survival (OS), univariate analysis of risk score calculated by model 1, gender, age, tumor stage, TNM stage, and Weiss score by using The Cancer Genome Atlas (TCGA)–ACC data. **(B)** Forest plot summary of analyses of OS, univariate analysis of risk score calculated by model 1, tumor stage, and M stage by using TCGA–ACC data. **(C)** Forest plot summary of analyses of OS, univariate analysis of risk score calculated by model 2, gender, age, tumor stage, TNM stage, and Weiss score by using TCGA-ACC data. **(D)** Forest plot summary of analyses of OS, univariate analysis of risk score calculated by model 2, tumor stage, and M stage by using TCGA-ACC data. HR, hazard ratio; OS, overall survival.

Because we had identified the two best models before, we respectively included the risk score of model 1 (or model 2) and important factors we mentioned before for the Cox regression analysis. Risk score of model 1 (hazard ratio = 4.822, 95%CI of ratio: 2.636–8.821, *P* < 0.001), risk score of model 2 (hazard ratio = 4.465, 95% CI of ratio: 2.449–8.143, *P* < 0.001), tumor stage (hazard ratio = 6.097, 95% CI of ratio: 2.433–15.282, *P* < 0.001), and M-stage (hazard ratio = 5.806, 95% CI of ratio: 2.471–13.640, *P* < 0.001) were influence features of OS as suggested by univariate Cox analysis (Figures 4A,C). In model 1, the results of multivariate Cox analysis suggested that even being adjusted by other features, risk scores of model 1 (hazard ratio = 4.663, 95% CI of ratio: 2.238–9.713, *P* < 0.001) were still relevant to OS among patients with ACC as shown in Figure 4B. In model 2, the results of multivariate Cox analysis suggested that even being adjusted by other features, risk scores of model 2 (hazard ratio = 5.603, 95% CI of ratio: 2.410–13.024, *P* < 0.001) and tumor stage (hazard ratio = 3.803, 95%CI of ratio: 1.223–11.822, *P* = 0.021) were still relevant to OS among patients with ACC (Figure 4D).

### Identification of Model 1 and Model 2-Associated Biological Pathways

With the cutoff criteria we set before, only one risk score-related KEGG signaling pathways were enriched in model 1 including cell cycle (nominal *P* < 0.001, | ES| = 0.738, gene size = 123, and FDR = 0.903%) as shown in Supplementary Table 6. In model 2, two risk score-related KEGG signaling pathways were significantly enriched including cell cycle (nominal *P* < 0.001, | ES| = 0.722, gene size = 123, and FDR = 0.884%) and oocyte meiosis (nominal *P* < 0.001, | ES| = 0.604, gene size = 111, and FDR = 0.913%; Supplementary Table 6). Interestingly, these two pathways were consistent with the results of KEGG analysis of genes in the blue module. We also increased the biological pathways related to C5 (ontology gene sets). The results indicated that model 1 was significantly associated with DNR replication-related and cell cycle-related signaling pathways (Supplementary Table 6). The top three enriched biological pathways were mitotic sister chromatid segregation (nominal P < 0.001, | ES| = 0.829, gene size = 158, and FDR = 0%), condensed chromosome centromeric region (nominal P < 0.001, | ES| = 0.827, gene size = 108, and FDR = 0%), and kinetochore (nominal P < 0.001, | ES| = 0.809, gene size = 128, and FDR = 0%). In addition, model 2 was enriched in 43 biological pathways (Supplementary Table 6), and the top three were mitotic sister chromatid segregation (nominal P < 0.001, | ES| = 0.824, gene size = 158, and FDR = 0%), condensed chromosome centromeric region (nominal P < 0.001, | ES| = 0.811, gene size = 108, and FDR = 0%), and sister chromatid segregation (nominal P < 0.001, | ES| = 0.803, gene size = 187, and FDR = 0%). C6 (oncogenic signature gene sets)-related biological pathways were also explored. We found that model 1 was significantly related to seven biological pathways. Meanwhile, model 2 was associated with six pathways (Supplementary Table 6). Both the two models were associated with CSR LATE UP.V1 UP, RB P107 DN.V1 UP, E2F1 UP.V1 UP, RPS14 DN.V1 DN, GCNP SHH UP LATE.V1 UP, and VEGF A UP.V1 DN.

### Model 2 Was Regarded as the Best Model With Better Clinical Utility

**FIGURE 5:**
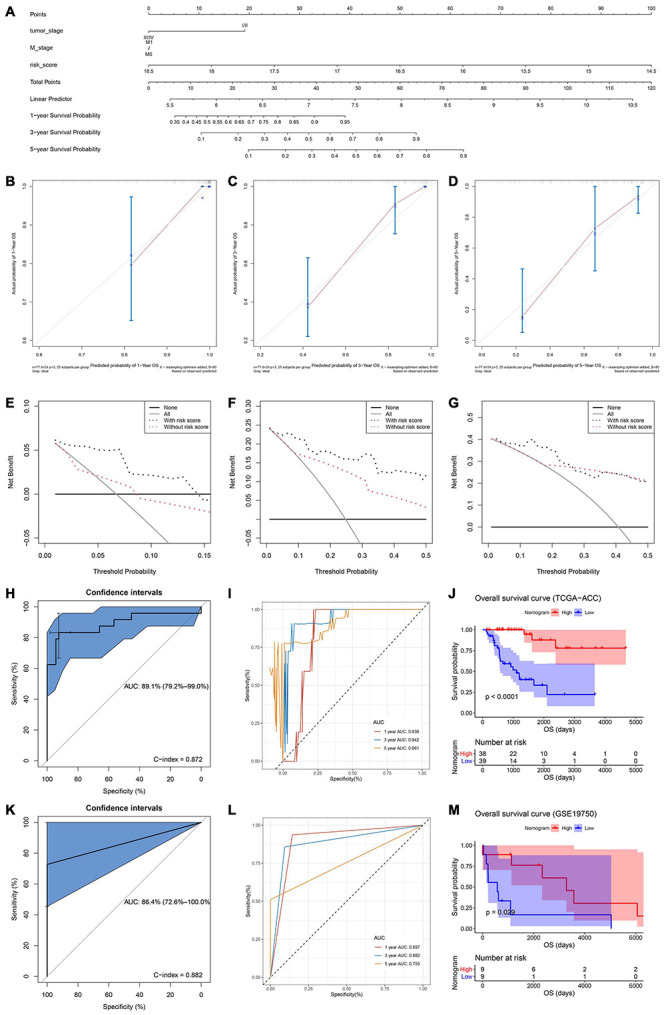
**(A)** The nomogram constructed with model 2 for predicting proportion of patients with 1-, 3-, or 5-year OS. The calibration plots for predicting 1- **(B)**, 3- **(C)**, or 5- **(D)** year OS. Decision curve analysis (DCA) for assessment of the clinical utility for 1- **(E)**, 3- **(F)**, or 5- **(G)** year OS of model 2, the x-axis represents the percentage of threshold probability, and the y-axis represents the net benefit. OS, overall survival; DCA, decision curve analysis. **(H)** Receiver operating characteristic (ROC) curves and area under the curve (AUC) statistics to evaluate the diagnostic efficiency of the nomogram based on model 2 in TCGA–ACC data. **(I)** Time-dependent ROC curves indicating the predictive accuracy of the nomogram based on model 2 for 1-, 3-, or 5-year OS based on TCGA–ACC data. **(J)** Survival analysis of the association between risk score calculated by model 2 and overall survival time in ACC using TCGA-ACC data. **(K)** Receiver operating characteristic (ROC) curves and area under the curve (AUC) statistics to evaluate the diagnostic efficiency of the nomogram based on model 2 in GSE19750. **(L)** Time-dependent ROC curves indicating the predictive accuracy of the nomogram based on model 2 for 1-, 3-, or 5-year OS based on GSE19750. **(M)** Survival analysis of the association between risk score calculated by model 2 and overall survival time in ACC using GSE19750.

Both of the two candidate models showed great prognostic values of patients with ACC according to previous analyses. In order to distinguish the best prognostic model, we further constructed two nomograms separately according to risk scores of model 1 (Supplementary Figure 9A) and model 2 (Figure 5A) and other significant features in multivariate Cox analysis. Both of the two models showed good potential for clinical application. According to the result of the calibration curves (Figures 5B–D and Supplementary Figures 9B-D), the nomogram of model 2 showed better value compared with the ideal model and model 1. Especially for nomogram’s 3-year and 5-year OS, the nomogram showed great performance almost as the same as the ideal model did (Figures 5C,D). Thus, we regarded model 2 (α = 0.6) as the best prognostic model in the present study.

### Validation of the Nomogram Assessed by the Best Model

In order to validate the predictive value of the nomogram based on the best model, we further performed DCA, C-index, and ROC analyses. DCA was performed to evaluate the clinical net benefit using the nomogram with and without risk score (calculated by the best model) for predicting 1-, 3-, and 5-year survival probability. As Figure 5E showed, there was a trend that the nomogram based on the best model occupied higher net benefit than the nomogram without risk score when predicting 1-year survival probability, when Pt ranged between 0 and 0.15. For the evaluation of the clinical net benefit using the nomogram based on the best model for predicting 3-year survival probability, the nomogram with the best model had higher net benefit compared with the nomogram without risk score, when Pt ranging from 0 to 0.50 (Figure 5F). The nomogram with the best model also obviously improved the net benefit for 5-year survival prediction. In detail, the nomogram with the best model had a higher net benefit than the simple nomogram of Pt between 0 to 0.20 (Figure 5G). Summarizing above, the nomogram assessed by the best model showed high potential for clinical application, especially for 1- and 3-year survival prediction. ROC analysis using TCGA-ACC data indicated that the nomogram could predict OS of ACC patients effectively (AUC: 0.891; C-index: 0.872; Figure 5H). Time-dependent ROC curves demonstrated that this nomogram with the best model showed excellent stability over a period of 5 years (1-year AUC: 0.838, 3-year AUC: 0.942, 5-year AUC: 0.961, Figure 5I). We directly used the nomogram to divide the ACC patients into high- and low-point groups and survival analysis demonstrated that ACC patients with higher nomogram points had better OS compared with patients with lower nomogram points (P < 0.0001, Figure 5J). We also validated the predication value of this nomogram by using GSE19750, we got the similar conclusion that the nomogram with the best model could predict OS of ACC patients effectively (AUC: 0.864; C-index: 0.882; Figure 5K). Time-dependent ROC curves demonstrated that this nomogram based on the best model showed good stability over a period of 5 years (1-year AUC: 0.897, 3-year AUC: 0.882, 5-year AUC: 0.755, Figure 5L). Survival analysis also demonstrated that ACC patients in highnomogram point group had better OS compared with ACC patients in low-nomogram point group (P = 0.029, Figure 5M).

### A Summary of Mutations and Copy Number Variations of Genes in the Best Model

**FIGURE 6:**
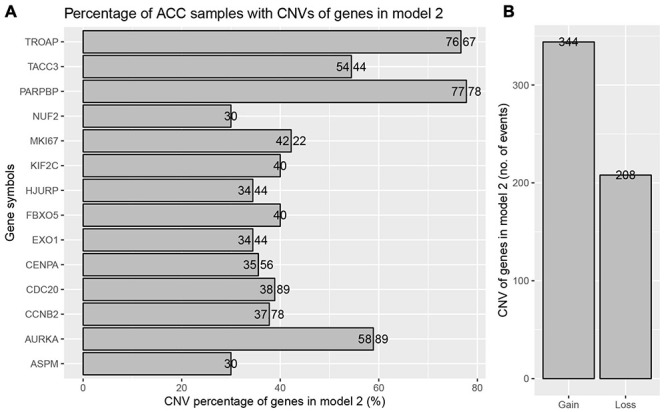
Copy number variations (CNVs) of genes in model 2 in ACC. **(A)** Percentage of ACC samples with CNVs of genes in model 2 based on TCGA-ACC data. **(B)** Events of copy number gain or loss of genes in model 2 in ACC samples.

The best model contained 14 most powerful prognostic markers [ASPM (abnormal spindle microtubule assembly), AURKA (aurora kinase A), CCNB2 (cyclin B2), CDC20 (cell division cycle 20), CENPA (centromere protein A), EXO1 (exonuclease 1), FBXO5 (F-box protein 5), HJURP (Holliday junction recognition protein), KIF2C (kinesin family member 2C), MKI67 (marker of proliferation Ki-67), NUF2 (NUF2 component of NDC80 kinetochore complex), PARPBP (PARP1 binding protein), TACC3 (transforming acidic coiled-coil containing protein 3), and TROAP (trophinin associated protein)], which were selected for further analysis. Among the 92 ACC patients with sequencing data, only in 10 independent samples contained mutations of genes as in the best model (Table 2). As for genetical alteration of the 14 genes, MKI67 altered the most (12%), and the main type was mRNA High (Supplementary Figure 10). Combined with the relative mRNA expression values of these genes, these genes seemed to highly express when there exist alterations in them (Supplementary Figure 10). Among the 90 ACC samples with CNV data, CNVs of the 14 genes constantly existed (Figure 6A and Table 3). Among the 14 genes, PARPBP was the most associated gene, which had the highest frequency of CNV events (77.78%, 70/90) (Figure 6A and Table 3). Moreover, gain of copy number was the most common CNV event (62.32%, 344/552, Figure 6B).

**TABLE 2:** Mutations of genes in model 2 in adrenocortical carcinoma (ACC) patients from The Cancer Genome Atlas (TCGA) database.

**ACC Sample ID**	**ASPM**	**AURKA**	**CDC20**	**EXO1**	**FBXO5**	**HJURP**	**KIF2C**	**MKI67**	**TACC3**
TCGA-PK-A5HB	I1717*								
TCGA-OU-A5PI	Q2415H								
TCGA-OR-A5K4	R3198C		V361I						
TCGA-OR-A5JA		V27M		C695F				D2208Y	D709Y
TCGA-OR-A5JC					L127Ifs*6				
TCGA-OR-A5L3						P459L			
TCGA-OR-A5K9							T575I		
TCGA-OR-A5JP								R2786Q	
TCGA-OR-A5LP								T284N	
TCGA-OR-A5JG								P3145H	

ACC, adrenocortical carcinoma. *a special mutation type of ASPM.

**TABLE 3:** Different copy number variation (CNV) patterns occur in ACC samples from TCGA database.

	**Diploid**	**Deep deletion**	**Shallow deletion**	**Copy number gain**	**Amplification**	**CNV sum**	**Percentage**
ASPM	63	0	13	13	1	27	30.00%
AURKA	37	0	3	49	1	53	58.89%
CCNB2	56	0	22	11	1	34	37.78%
CDC20	55	0	32	2	1	35	38.89%
CENPA	58	0	18	14	0	32	35.56%
EXO1	59	0	15	16	0	31	34.44%
FBXO5	54	0	19	16	1	36	40.00%
HJURP	59	2	16	13	0	31	34.44%
KIF2C	54	0	32	3	1	36	40.00%
MKI67	52	0	10	28	0	38	42.22%
NUF2	63	0	11	14	2	27	30.00%
PARPBP	20	1	6	63	0	70	77.78%
TACC3	41	0	10	37	2	49	54.44%
TROAP	21	0	1	65	3	69	76.67%

### Relationship Between Alterations in Genes in the Best Model and the Clinical Features

Based on the data from TCGA database, six clinical features (gender, age, pathologic stage, T stage, N stage, and M stage) were collected for this analysis. As the result suggested (Table 4), alterations in genes were significantly associated with pathologic stage (Chi-square = 10.644, *P* = 0.014), T stage (Chi-square = 11.008, *P* = 0.012), and M stage (Chi-square = 10.687, *P* = 0.001).

**TABLE 4:** Clinical pathological parameters of ACC patients with or without mutation/CNV of genes in model 2.

		**With mutation and/or CNV**	**Without mutation and CNV**	**Total number**	**Chi-square**	***P*-value**
Age	≥65	6	8	14	0.200	0.655
	<65	24	50	74		
Gender	Female	19	40	59	0.284	0.594
	Male	11	18	29		
Pathologic stage	I	3	6	9	10.644	0.014
	II	10	33	43		
	III	5	13	18		
	IV	12	6	18		
T stage	T1	3	6	9	11.008	0.012
	T2	11	37	48		
	T3	3	8	11		
	T4	13	7	20		
N stage	N0	24	54	78	2.195	0.138
	N1	6	4	10		
	Nx	0	0	0		
M stage	M0	18	52	70	10.687	0.001
	M1	12	6	18		
	Mx	0	0	0		

*With mutation or CNV: Cases have mutant or CNV or mutant + CNV, confirmed through TCGA database. Without mutation and CNV: Cases with neither mutant nor CNV, confirmed through TCGA database.*

### The Effects of Alterations in the 14 Genes on the Relative mRNA Expression Levels

**FIGURE 7:**
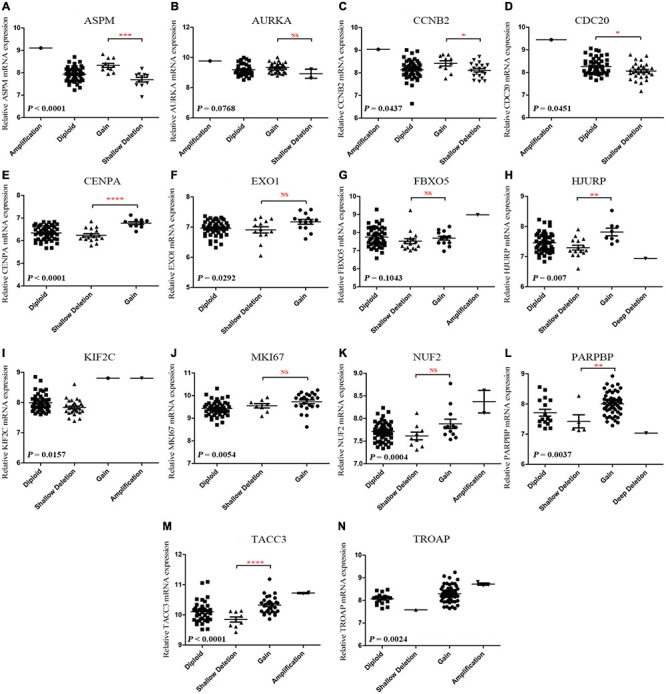
**(A-N)** Correlation between different CNV patterns and mRNA expression levels of genes in model 2, respectively.

According to the result, ASPM (P < 0.0001), CCNB2 (P = 0.0437), CDC20 (P = 0.0451), CENPA (P < 0.0001), EXO1 (P = 0.0292), HJURP (P = 0.007), KIF2C (P = 0.0157), MKI67 (P = 0.0054), NUF2 (P = 0.0004), PARPBP (P = 0.0037), TACC3 (P < 0.0001), and TROAP (P = 0.0024) were differently expressed among different types of CNVS. In detail, copy number gains of ASPM (P < 0.001, Figure 7A), CCNB2 (P < 0.05, Figure 7C), CENPA (P < 0.0001, Figure 7E), HJURP (P < 0.01, Figure 7H), PARPBP (P < 0.01, Figure 7L), and TACC3 (P < 0.0001, Figure 7M) were significantly associated with higher mRNA expression comparing with these in copy number shallow deletions (or deep deletions). As for AURKA (Figure 7B), EXO1 (Figure 7F), FBX05 (Figure 7G), MKI67 (Figure 7J), and NUF2 (Figure 7K), there was no significant expression difference between gains of copy number and shallow deletions of copy number. There was no copy number gain in CDC20, but samples with shallow deletion were significantly lower expressed compared with the diploid ones (P = 0.0451, Figure 7D). There were only one sample with copy number gain on KIF2C (Figure 7I) and one sample with copy number with shallow deletion of TROAP (Figure 7N), which perhaps meant that the result might not be convincing.

### Correlation Between Copy Number Variations of the 14 Genes and Survival of Patients With Adrenocortical Carcinoma

**FIGURE 8:**
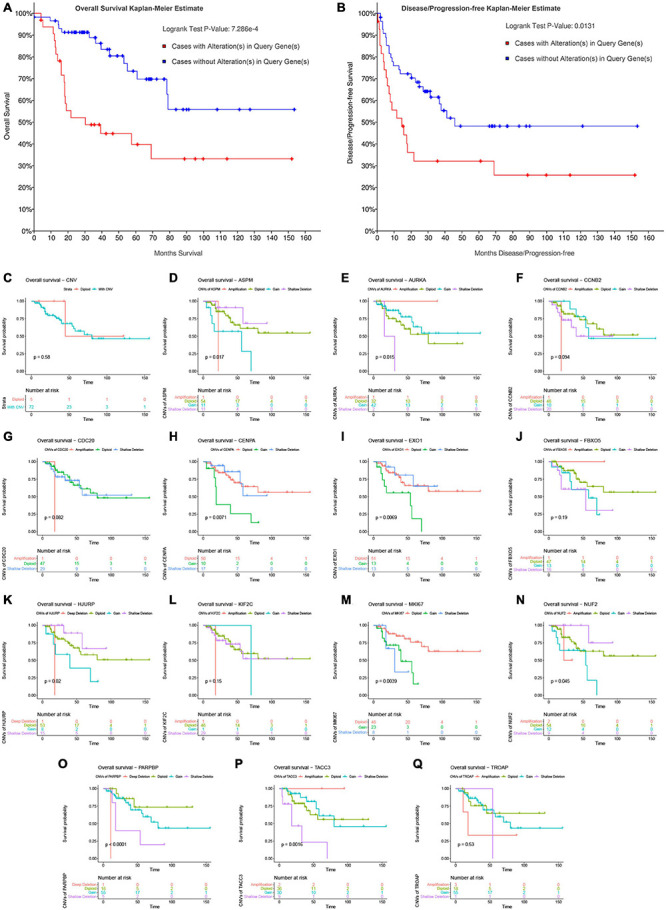
Survival analysis of ACC patients with CNVs of genes in model 2 based on TCGA-ACC data. Overall survival **(A)** and disease-free survival **(B)** of patients with alterations in genes in model 2 or patients without alterations in genes in model 2. **(C)** Overall survival of patients with any CNVs of genes in model 2 or patients with diploid. Overall survival of patients with different CNV types of ASPM **(D)**, AURKA **(E)**, CCNB2 **(F)**, CDC20 **(G)**, CENPA **(H)**, EXO1 **(I)**, FBXO5 **(J)**, HJURP **(K)**, KIF2C **(L)**, MKI67 **(M)**, NUF2 **(N)**, PARPBP **(O)**, TACC3 **(P)**, and TROAP **(Q)**.

In this step, survival analysis was performed to explore the prognostic value of CNVs in the 14 genes. According to the result, ACC patients with alterations in the 14 genes had poor OS (*P* = 7.286E−4, Figure 8A) and DFS (*P* = 0.0131, Figure 8B). Unfortunately, there was no association between patients with CNV or without CNV and OS (*P* = 0.58, Figure 8C). Then we separately explored the association between OS in each gene and CNVs. The result demonstrated that shallow deletions in ASPM (*P* = 0.0170, Figure 8D), CENPA (*P* = 0.0071, Figure 8H), EXO1 (*P* = 0.0069, Figure 8I), HJURP (*P* = 0.0200, Figure 8K), and NUF2 (*P* = 0.0450, Figure 8N) led to better OS of patients with ACC compared with those being affected by copy number gains, while patients with shallow deletions in AURKA (*P* = 0.0150, Figure 8E), MKI67 (*P* = 0.0039, Figure 8M), PARPBP (*P* < 0.0001, Figure 8O), and TACC3 (*P* = 0.0016, Figure 8P) had poorer OS. As for MKI67, patients affected by CNVs (shallow deletion or gain) had poorer OS compared with those affected by diploid (*P* = 0.0039, Figure 8M). As for CCNB2 (*P* = 0.094; Figure 8F), CDC20 (*P* = 0.062, Figure 8G), FBXO5 (*P* = 0.190, Figure 8J), and KIF2C (*P* = 0.150, Figure 8L), there were no significantly survival difference between different CNVs.

### Validation of the 14 Genes in the Best Model

According to the result, all the classifiers constructed and verified by TCGA–ACC data showed good performance because the accuracy of classification was more than 0.70 (Table 5). Especially the polynomial-SVM-based classifier, its accuracy was 0.9870, and we validated all the classifiers by using GSE19750, GSE76019, and GSE76021. All the classifiers showed good performance in GSE76019 and GSE76021 (accuracy of classification ≥ 0.50, Table 5), but in GSE19750, KNN-based classifier and all the SVM-based classifiers did not play well as we expected (Table 5), perhaps because only 22 ACC samples from GSE19750 were included for validation. All in all, all the classifiers constructed based on the 14 genes showed great performance to distinguish dead ACC samples from live ACC samples, which meant all the 14 genes were significantly associated with prognosis of patients with ACC.

**TABLE 5:** The accuracy of classification of linear discriminant analysis (LDA)-based classifier, K-nearest neighbor (KNN)-based classifier, linear-support vector machine (SVM)-based classifier, polynomial-SVM-based classifier, radial basis function (RBF)-SVM-based classifier, and sigmoid-kernel-SVM-based classifier.

		**LDA**	**KNN**	**Kappa**	**Linear-SVM**	**Kappa**	**Polynomial-SVM**	**Kappa**	**RBF-SVM**	**Kappa**	**Sigmoid-kernel SVM**	**Kappa**
TCGA-ACC	Training set	0.8701	0.8961	0.7463	0.8831	0.7111	0.9870	0.9694	0.9351	0.8469	0.8182	0.5664
GSE19750	Testing set 1	0.6364	0.2727	−0.0353	0.4091	0.0403	0.1818	0	0.2727	0.0435	0.4545	0.0704
GSE76019	Testing set 2	0.7647	0.7059	0.2056	0.7059	0.3561	0.6471	0	0.7353	0.3319	0.7647	0.5211
GSE76021	Testing set 3	0.6207	0.7241	0.7241	0.7931	0.5272	0.6897	0.2162	0.5862	−0.0235	0.7931	0.5272

*LDA, Linear discriminant analysis; KNN, K-Nearest Neighbor; RBF, radial basis function; SVM, Support Vector Machine.*

### Hub Genes in the Best Model

All the 14 genes from the best model were included in this PPI network (Supplementary Figure 11A). As shown in Supplementary Figure 11B, the degrees of six genes (ASPM, AURKA, CCNB2, CDC20, KIF2C, and NUF2) were higher than any other of the remaining eight genes (Degree = 13). Thus, the six genes were considered as hub genes in the best model. Expression of ASPM was significantly related to CCNB2, CDC20, FBXO5, HJURP, MKI67, NUF2, PARPBP, TACC3, and TROAP (Supplementary Figure 11C). Among them, MKI67 was the most related gene (Supplementary Figure 11D). Expression of AURKA was significantly associated with FBXO5, HJURP, MKI67, PARPBP, TACC3, and TROAP (Supplementary Figure 11C). Moreover, PARPBP was best associated with AURKA (Supplementary Figure 11D). Expression of CCNB2 was relevant to ASPM, CDC20, CENPA, FBXO5, HJURP, and PARPBP (Supplementary Figure 11C), and PARPBP was the most associated gene with CCNB2 (Supplementary Figure 11D). Expression of CDC20 was associated with CCNB2, ASPM, FBXO5, HJURP, KIF2C, MKI67, PARPBP, and TROAP (Supplementary Figure 11C), and the most related gene was HJURP (Supplementary Figure 11D). The expression of NUF2 was significantly related to HJURP, FBXO5, CENPA, CDC20, AURKA, and ASPM (Supplementary Figure 11C). Among them, ASPM was the most associated gene (Supplementary Figure 11D).

### External Validation of the Best Model/the Nomogram and Comparison With Other Biomarkers Using Gene Expression Data

In order to assess the prognostic value of the six hub genes, and compare the prognostic value of the best model/the nomogram with the prognostic biomarkers given by other studies, we collected several biomarkers from previous studies. In total, eight biomarkers including CTNNB1 (Assié et al., 2014), IGF2 (Catalano et al., 2021), TP53 (Assié et al., 2014), MKI67 (Beuschlein et al., 2015), SF1 (Tian et al., 2021), IPRPs model (Tian et al., 2021), m6A-related signature (Jin et al., 2021), and m6A-based signature (Shen et al., 2021) were used in the present study. According to the result (Table 6), all the hub genes could effectively predict the OS of ACC patients (ASPM: AUC = 0.840, C-index = 0.827; AURKA: AUC = 0.786, C-index = 0.794; CCNB2: AUC = 0.830, C-index = 0.821; CDC20: AUC = 0.833, C-index = 0.838; KIF2C: AUC = 0.819, C-index = 0.823; NUF2: AUC = 0.811, C-index = 0.843). Moreover, the risk score calculated by the best model (AUC = 0.844, C-index = 0.851) and the nomogram based on the best model (AUC = 0.891, C-index = 0.872) showed better ability for predicting prognosis than those biomarkers collected from previous studies. AUC and C-index of these biomarkers are shown in Table 6. To summarize the above, the results demonstrated that the best model and the nomogram based on the best model may act better than other biomarkers.

**TABLE 6:** Comparison the best model vs. the nomogram with other biomarkers using TCGA–ACC data.

**Source**	**Biomarker evaluation**	**AUC**	**C-index**
	ASPM	0.840	0.827
	AURKA	0.786	0.794
	CCNB2	0.830	0.821
	CDC20	0.833	0.838
	KIF2C	0.819	0.823
	NUF2	0.811	0.843
	Risk score (a = 0.6)	0.844	0.851
	Nomogram	0.891	0.872
PMID: 24747642	CTNNB1	0.657	0.613
PMID: 33075426	IGF2	0.562	0.598
PMID: 24747642	TP53	0.565	0.603
PMID: 25559399	MKI67	0.818	0.859
PMID: 33626208	SF1	0.635	0.621
PMID: 33626208	IPRPs model	0.772	0.806
PMID: 33746977	m6A-related signature	0.818	0.778
PMID: 33692753	m6A based signature	0.744	0.696

## Discussion

Adrenocortical carcinoma is a relatively rare malignancy in the urinary system associated with a poor prognosis (Lam, 2021). 5-year survival rate for patients with ACC was 50% as ASCO reported. Similarly, it is not optimistic that 40% of the cases have distant metastasis when diagnosed (Ayala-Ramirez et al., 2013; Guillaume et al., 2014), which means that they have missed the best opportunity for treatment. Thus, there is of great need to develop novel molecular biomarkers for diagnosis and prognosis of ACC patients.

Over the past several years, the discoveries of novel and informative genes by using bioinformatics methods have provided valuable information in the diagnosis of malignancies (He et al., 2017; Meulekamp et al., 2017). At present, WGCNA has been widely used to screen out novel and effective molecular biomarkers in the bioinformatics field (Gu et al., 2019; Li et al., 2019; Lv et al., 2021). In our previous study, we have made great efforts to use WGCNA as the main method to identify prognosis biomarkers for bladder cancer (Yan et al., 2019). Thus, we first identified genes associated with survival of ACC in the present study, and 93 biomarkers significantly relevant to survival of ACC were further identified combined with overall survival analysis and disease-free survival analysis. Interestingly, some studies thought that a single biomarker might not predict the prognosis of patients with ACC well (Chen et al., 2019). Nowadays, more and more studies have paid their attention on developing a multi-gene signature for predicting prognosis in malignancies instead of just a single biomarker (Qiao et al., 2019; Wu et al., 2019). Given that the Ridge, Elastic Net, and Lasso methods have been widely used to construct a Cox proportional hazard regression model (Lu et al., 2019), 11 multiple-gene signatures were constructed for predicting prognosis of ACC patients. Two candidate models were further screened out and validated by calculating the AUC by using internal validation set. To test whether the prognostic value of the two signatures were independent of other clinical features (gender, age, tumor stage, T stage, N stage, M stage, and Weiss score), we included these factors for the Cox regression analysis. The result indicated that tumor stage, risk score of model 1, and risk score of model 2 were significant prognostic factors for ACC patients. Moreover, risk score of model 1 and risk score of model 2 were independent of tumor stage. Tumor stage including TNM staging system reflects the internal characteristics of tumors, which is of great value for evaluation of the degree of tumor deterioration and prediction of prognosis according to previous findings (Veeratterapillay et al., 2012). In our study, model 1 and model 2 could not only discriminate between low- and high-risk groups like tumor stage did, but also showed more effective prognostic value compared with tumor stage, which made our results reliable. To better understand the biological role of the two signatures, we performed GSEA analysis. Two KEGG pathways associated with model 1 were identified including cell cycle and oocyte meiosis. Meanwhile, one KEGG pathway associated with model 2 was identified including cell cycle. Cell cycle was the basic process of cell proliferation. According to a study by Kaistha et al. (2015), the deregulation of cell cycle was obviously associated with tumor progression. As for oocyte meiosis, maturation and development of oocyte is a complex biologic process in mammals, which is the key event in the reproductive process (Jaffe and Egbert, 2017). Any errors in this process can lead to the oocyte developmental abnormalities or infertility (Jaffe and Egbert, 2017). Meiosis is specialized cell division with the reduction in genetic content, which guaranteed the stability of chromosome numbers of gamogenetic organisms (Jaffe and Egbert, 2017). Subsequent analysis demonstrated that model 2 showed better predictive value than model 1; thus, we regarded model 2 as the best model in the present study. Moreover, the best model was validated to show strong association with survival of ACC patients and high accuracy for OS probability prediction in patients with ACC.

By exploring the association between mutations and CNVs with genes in the best model, we found that high expression levels of genes in the best model was associated with gene alteration. Subsequent analysis indicated that shallow deletions in ASPM, CENPA, EXO1, HJURP, and NUF2 caused better OS of ACC patients. Meanwhile, shallow deletions in AURKA, MKI67, PARPBP, and TACC3 led to poorer OS. To summarize the above, we found that alterations in these biomarkers might have strong effects on the survival of ACC patients. A PPI network was constructed based on the 14 genes from the best model; six hub genes were further identified according to the degree of connectivity. All the hub genes could effectively predict the OS of ACC patients. In the past few years, some potential prognostic biomarkers for ACC were identified. Tian et al. constructed a robust prognostic model for ACC (Tian et al., 2021). Two m6A-related signatures, which might be used as independent prognostic factors in evaluating the prognosis of ACC patients, were also established (Jin et al., 2021; Shen et al., 2021). Among the six hub genes we identified, two, including ASPM (Yuan et al., 2018) and CCNB2 (Gao et al., 2019; Guo et al., 2020), were already regarded as prognostic biomarkers in previous studies. Yuan et al. screened out 12 hub genes including ASPM, which were associated with the progression and prognosis of ACC. Two recent studies proved that CCNB2 was associated with worse OS of ACC (Gao et al., 2019; Guo et al., 2020). As for the rest of the four genes, the relationship between them and prognosis of ACC has never been reported by previous studies, which might be novel prognostic biomarkers for ACC. Moreover, the risk score calculated by the best mode and the nomogram based on the best model showed better ability in predicting prognosis than these biomarkers collected from previous studies.

There still exist some limitations in our study. First, though our study was a relatively preliminary exploration of establishing a multi-gene signature by combing bioinformatics methods, statistics, and machine learning, there might be lack of novelty in methods. Therefore, we will explore more novel bioinformatics and machine learning methods to integrate prognostic gene signatures. Second, the best prognostic model was developed and validated by using data derived from TCGA and GEO databases. Although the signature could distinguish high- and low-risk groups well, it was not clear if it could show good performance as we expected in clinical trials. Thus, we will further apply and test this signature in clinical judgment for prognosis of ACCs. Third, according to the results of DCA, the best signature showed high potential for 5-year survival prediction when 0.00 < Pt < 0.20, but when Pt equaled other values, this signature did not play well as we expected, perhaps due to the small size of these datasets. Therefore, we will further validate the multi-gene signature in larger and richer datasets.

To sum up, 93 hub genes in the co-expression network were identified as survival associated genes. Moreover, we constructed an Elastic Net model including 14 genes, which were validated to show good performance by using three independent datasets. This signature could act as an effective prediction tool for the prognosis of ACC patients independently. The best model-based nomogram was established based on risk score assessed by this risk signature to provide clinical doctors with a visual tool. Moreover, six hub genes were identified among the 14 genes from the best model, which might be novel prognostic biomarkers of prognosis of patients with ACC. However, the best model and six hub genes needs to be verified by using more novel bioinformatics methods, clinical trials, and larger datasets.

## Data Availability Statement

Publicly available datasets were analyzed in this study. This data can be found here: The datasets presented in this study can be found in online repositories. The names of the repository/repositories and accession number(s) can be found in the article/Supplementary Material.

## Author Contributions

SL and T-ZL conceived and designed the study. XY, Z-XG, D-HY, and X-PL performed the analysis procedures. XY, Z-XG, CC, and X-PL analyzed the results. SL, Z-WY, and T-ZL contributed analysis tools. XY contributed to the writing of the manuscript. All authors reviewed the manuscript.

## Conflict of Interest

The authors declare that the research was conducted in the absence of any commercial or financial relationships that could be construed as a potential conflict of interest.

## Footnotes

1 http://www.ncbi.nlm.nih.gov/geo/

2 http://www.cbioportal.org/

3 https://cytoscape.org/

## Supplementary Material

Supplementary Figure 1 Flow chart indicating the process used to select target genes included in the analysis.

Supplementary Figure 2 **(A)** Samples clustering to detect outliers (GSE76019). **(B)** The clustering was based on the expression data of GSE76019. The top 2,500 genes with the highest SD values were used for the analysis by WGCNA. The color intensity was proportional to tumor stage, survival years (survival time), and survival status.

Supplementary Figure 3 Determination of soft-thresholding power in the weighted gene co-expression network analysis (WGCNA). **(A)** Analysis of the scale-free fit index for various soft-thresholding powers (β). **(B)** Analysis of the mean connectivity for various soft-thresholding powers. **(C)** Histogram of connectivity distribution when β = 5. **(D)** Checking the scale free topology when β = 5.

Supplementary Figure 4 The cluster dendrogram of genes in GSE76019. Each branch in the figure represents one gene, and every color below represents one co-expression module.

Supplementary Figure 5 **(A)** Interaction relationship analysis of co-expression genes. Different colors of horizontal axis and vertical axis represent different modules. The brightness of yellow in the middle represents the degree of connectivity of different modules. There was no significant difference in interactions among different modules, indicating a high-scale independence degree among these modules. **(B)** Classical MDS plot whose input is the TOM dissimilarity. Each dot (gene) is colored by the module assignment.

Supplementary Figure 6 Bioinformatics analysis of genes in the hub modules. **(A)** Biological process of genes in the red module. **(B)** Biological process of genes in the blue module. **(C)** KEGG pathway enrichment of genes in the blue module.

Supplementary Figure 7 Ten-fold cross-validation for tuning parameter selection in the Ridge **(A)**, ELASTIC-NET with α varying from 0.1 to 0.9 **(E–J)**, and lasso model **(K)**. The solid vertical lines are the partial likelihood deviance SE. The dotted vertical lines are drawn at the optimal values by minimum criteria and 1-SE criteria.

Supplementary Figure 8 Distribution of risk scores of Ridge **(A)**, ELASTIC-NET with α varying from 0.1 to 0.9 **(E–J)**, and lasso model **(K)** of ACC patients based on TCGA-ACC data. The number of survivors and non-survivors with different risk scores based on TCGA-ACC data; red represents the number of non-survivors, and blue represents the number of survivors.

Supplementary Figure 9 **(A)** The nomogram constructed with model 1 for predicting proportion of patients with 1-, 3- or 5-year OS. The calibration plots for predicting 1- **(B)**, 3- **(C)** or 5- **(D)** year OS. OS: overall survival.

Supplementary Figure 10 Genetic alterations associated with genes in model 2 and expression heatmap of genes in model 2 based on the data from TCGA.

Supplementary Figure 11 Interaction among genes in model 2 and spearman correlation analysis of the genes in model 2. **(A)** Protein-protein interaction (PPI) network of genes in model 2. **(B)** Degree of connectivity of genes in model 2 according to the PPI network. **(C)** Spearman correlation analysis of the genes in model 2. **(D)** A visual summary for the spearman correlation analysis (scatter plot, bar plot of expression levels of genes in model 2, and spearman correlation coefficients).

Supplementary Table 1 Clinical characteristics of patients with ACC in each study.

Supplementary Table 2 GO biological processes of genes in blue module.

Supplementary Table 3 Survival analysis of hub genes in co-expression network.

Supplementary Table 4 The corresponding regression coefficients of each gene in models constructed by the Ridge, ELASTIC-NET, and LASSO methods.

Supplementary Table 5 Gene set enrichment analysis of model 1 and model 2 by using GSE76019.
