# Supplementary figures and images for: Identification and Validation of a Novel Prognosis Prediction Model in Adrenocortical Carcinoma by Integrative Bioinformatics Analysis, Statistics, and Machine Learning
Source: Front Cell Dev Biol. 2021 Jun 7;9:671359. doi: 10.3389/fcell.2021.671359 (PMC8215582; doi:10.3389/fcell.2021.671359)

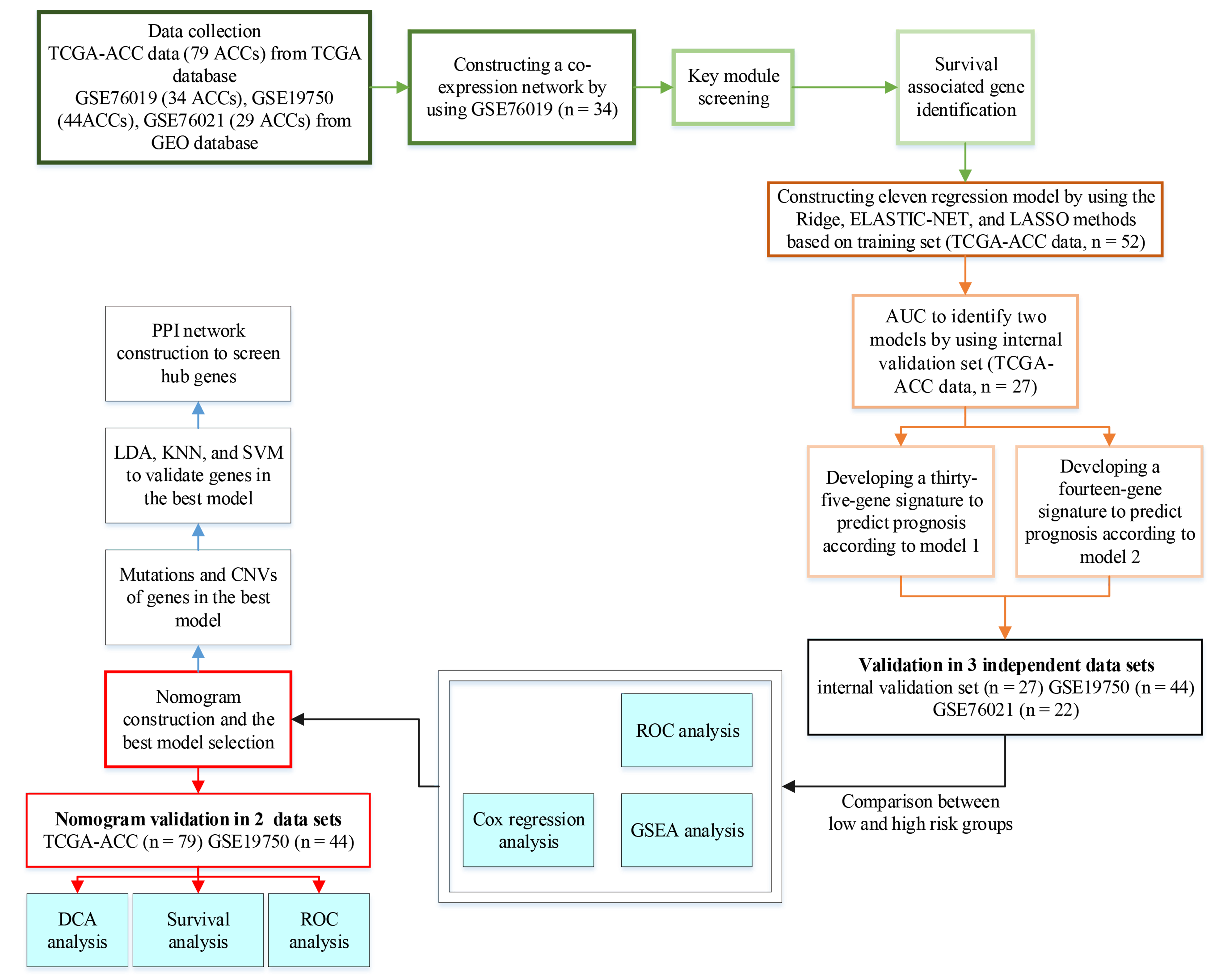

Supplement: Supplementary Figure 1 — Flow chart indicating the process used to select target genes included in the analysis. [file Image_1.TIF]

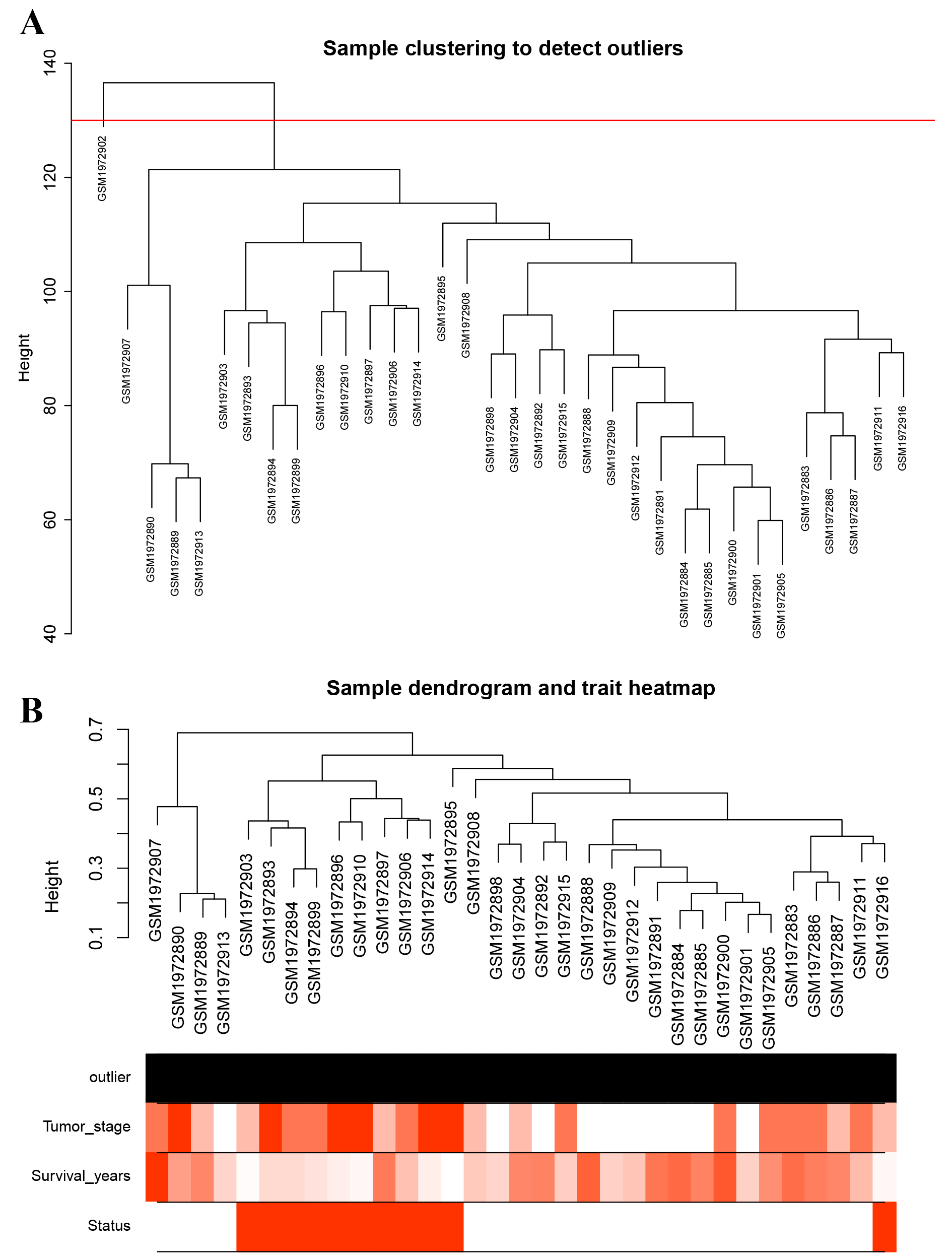

Supplement: Supplementary Figure 2 — (A) Samples clustering to detect outliers (GSE76019). (B) The clustering was based on the expression data of GSE76019. The top 2,500 genes with the highest SD values were used for the analysis by WGCNA. The color intensity was proportional to tumor stage, survival years (survival time), and survival status. [file Image_2.TIF]

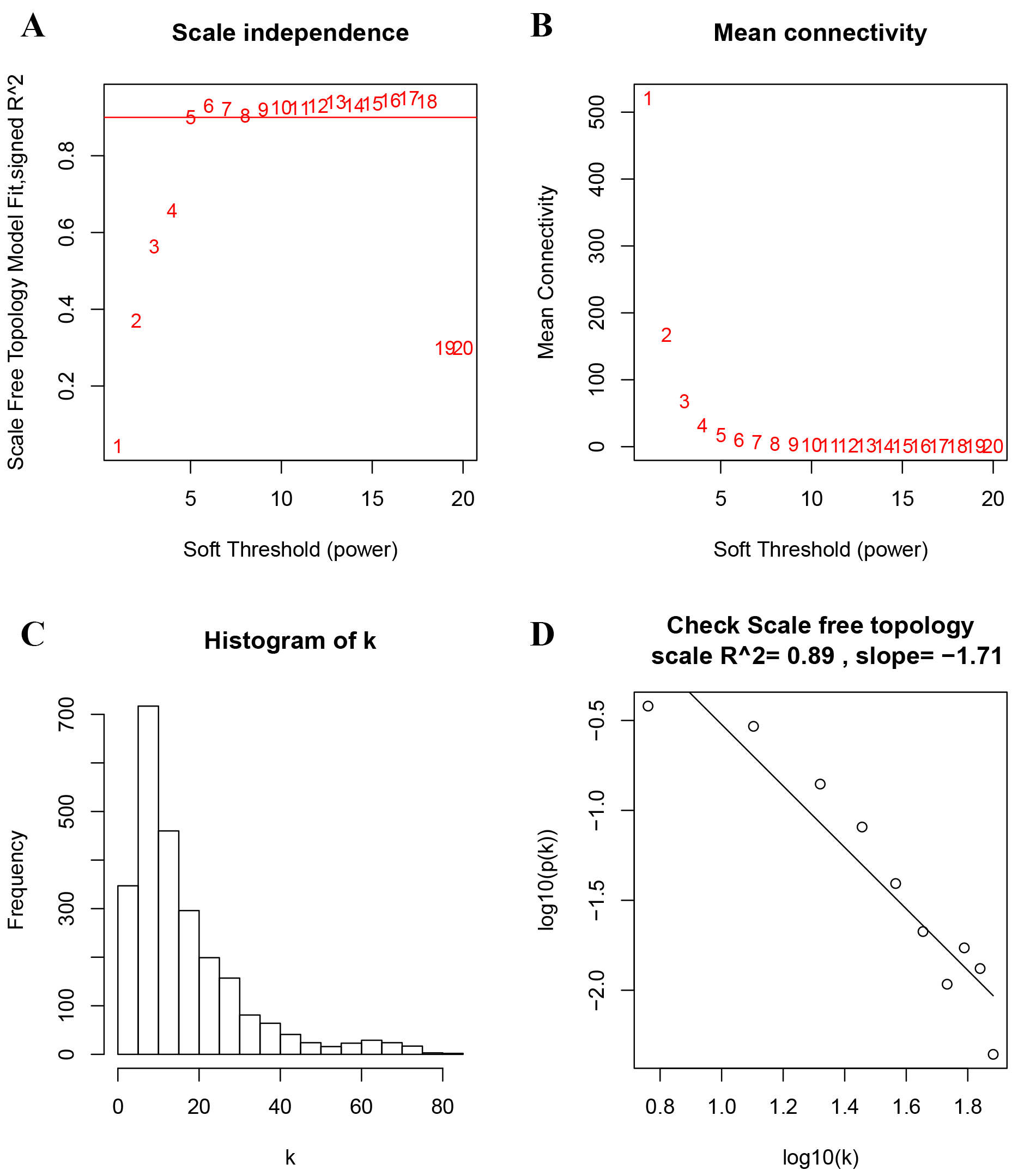

Supplement: Supplementary Figure 3 — Determination of soft-thresholding power in the weighted gene co-expression network analysis (WGCNA). (A) Analysis of the scale-free fit index for various soft-thresholding powers (β). (B) Analysis of the mean connectivity for various soft-thresholding powers. (C) Histogram of connectivity distribution when β = 5. (D) Checking the scale free topology when β = 5. [file Image_3.TIF]

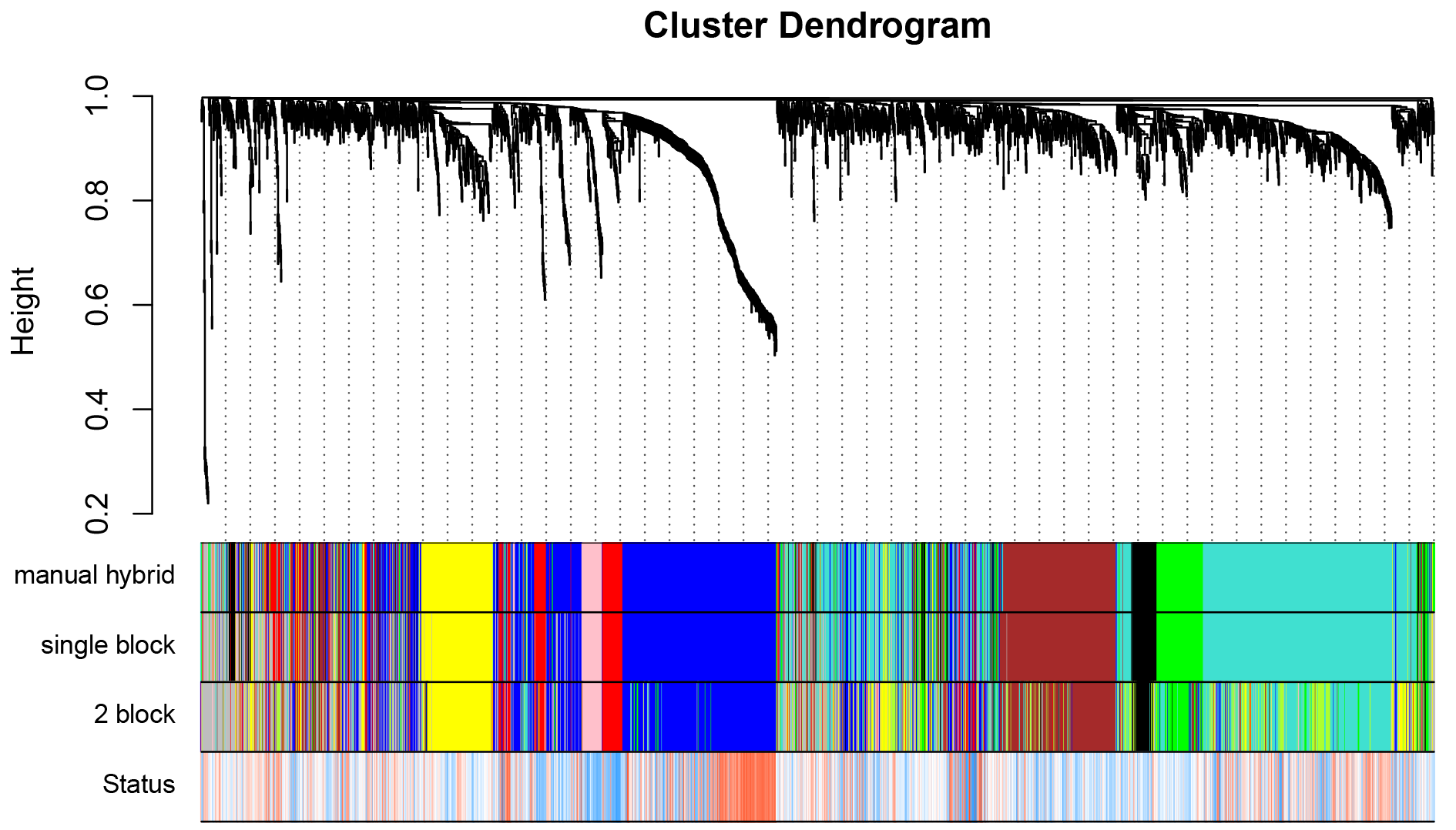

Supplement: Supplementary Figure 4 — The cluster dendrogram of genes in GSE76019. Each branch in the figure represents one gene, and every color below represents one co-expression module. [file Image_4.TIF]

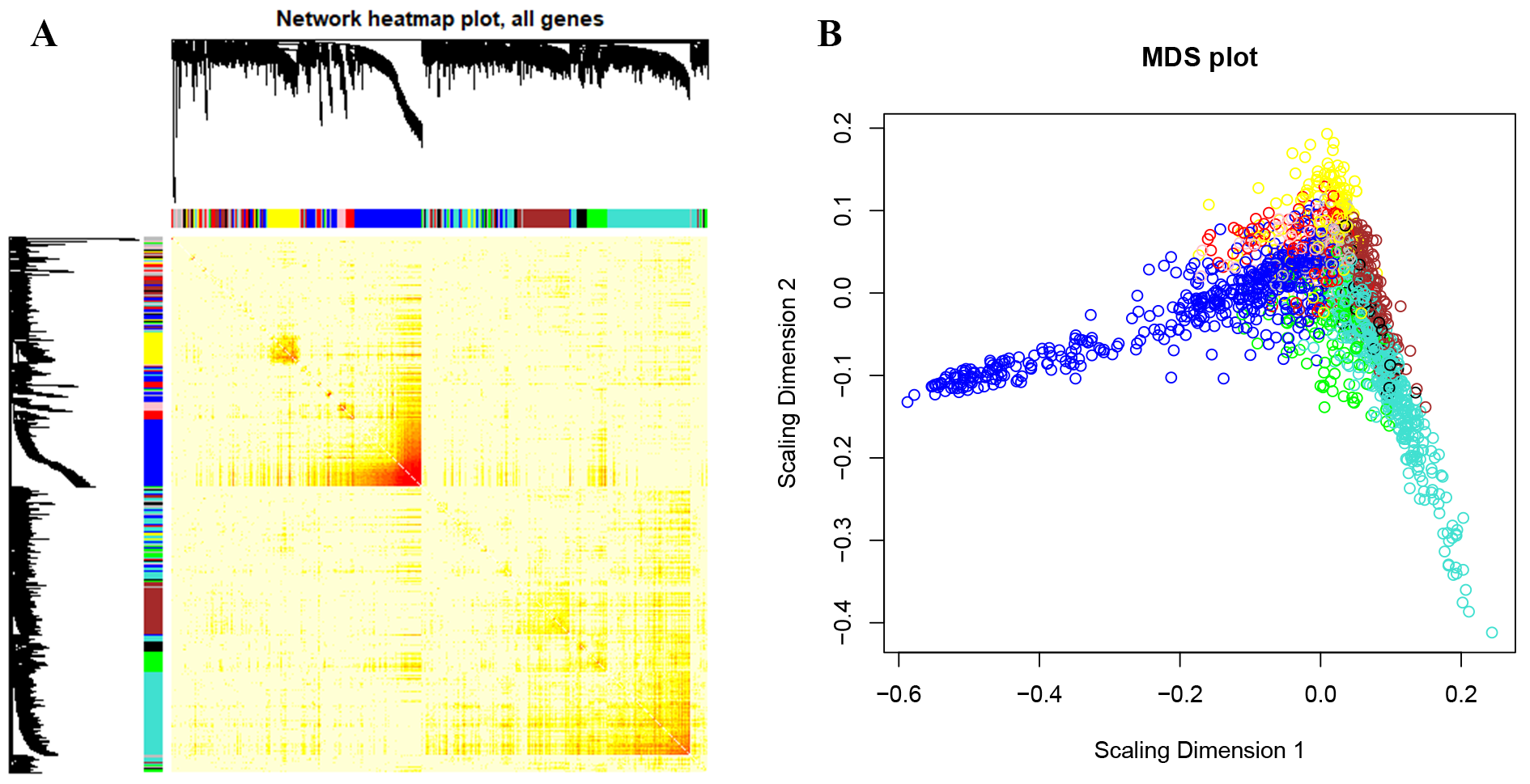

Supplement: Supplementary Figure 5 — (A) Interaction relationship analysis of co-expression genes. Different colors of horizontal axis and vertical axis represent different modules. The brightness of yellow in the middle represents the degree of connectivity of different modules. There was no significant difference in interactions among different modules, indicating a high-scale independence degree among these modules. (B) Classical MDS plot whose input is the TOM dissimilarity. Each dot (gene) is colored by the module assignment. [file Image_5.TIF]

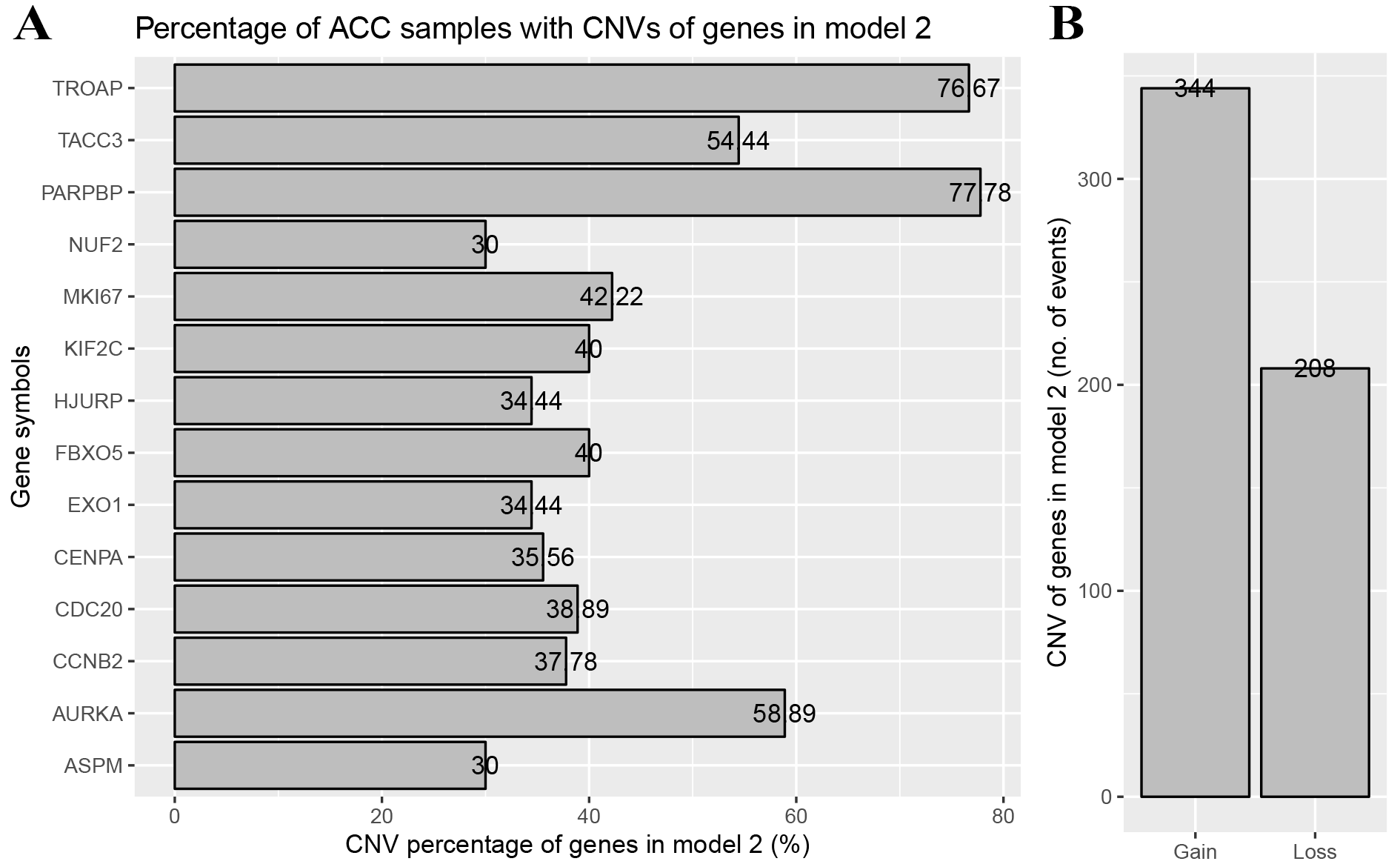

Supplement: Supplementary Figure 6 — Bioinformatics analysis of genes in the hub modules. (A) Biological process of genes in the red module. (B) Biological process of genes in the blue module. (C) KEGG pathway enrichment of genes in the blue module. [file Image_6.TIF]

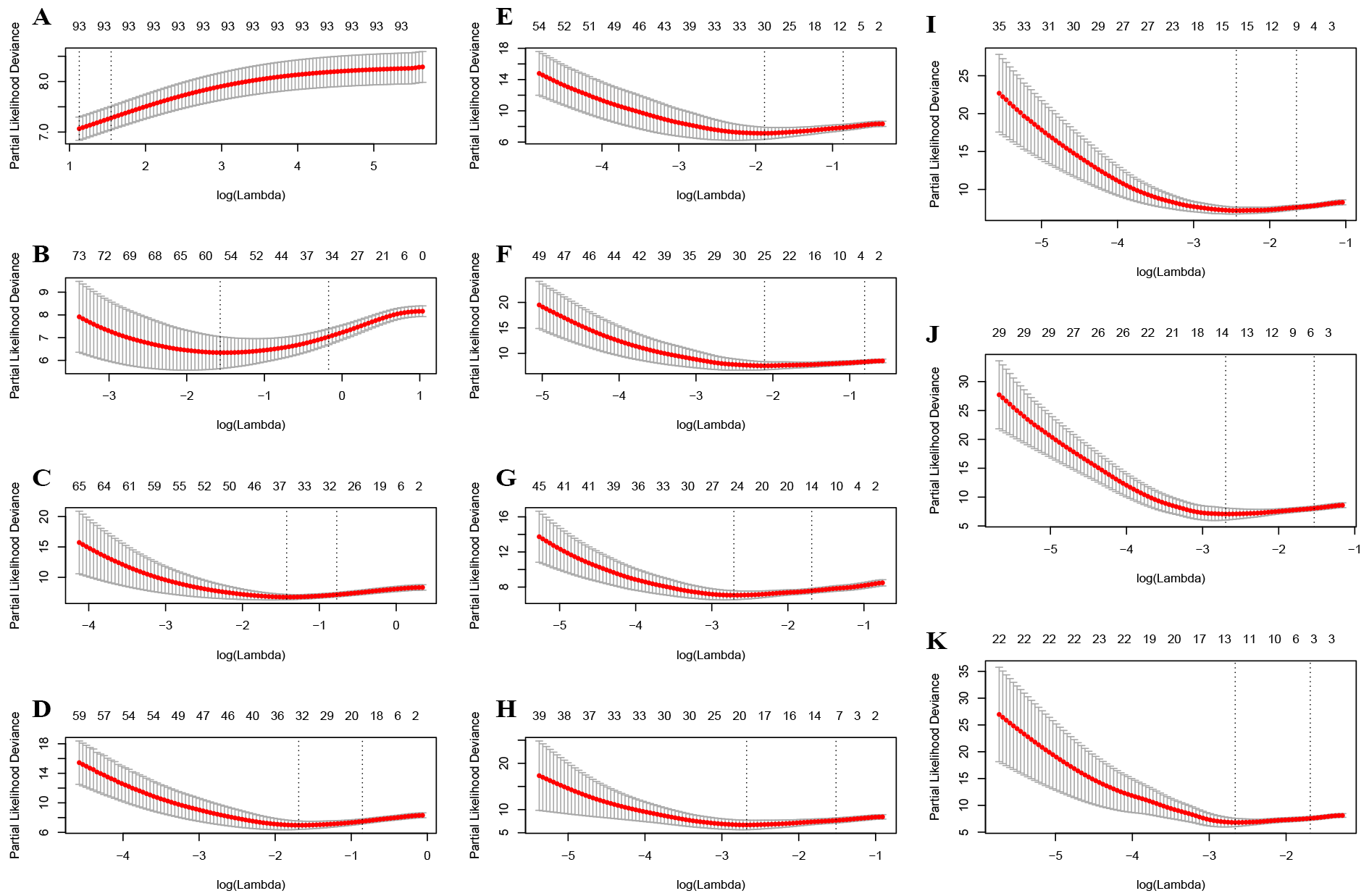

Supplement: Supplementary Figure 7 — Ten-fold cross-validation for tuning parameter selection in the Ridge (A), ELASTIC-NET with α varying from 0.1 to 0.9 (E–J), and lasso model (K). The solid vertical lines are the partial likelihood deviance SE. The dotted vertical lines are drawn at the optimal values by minimum criteria and 1-SE criteria. [file Image_7.TIF]

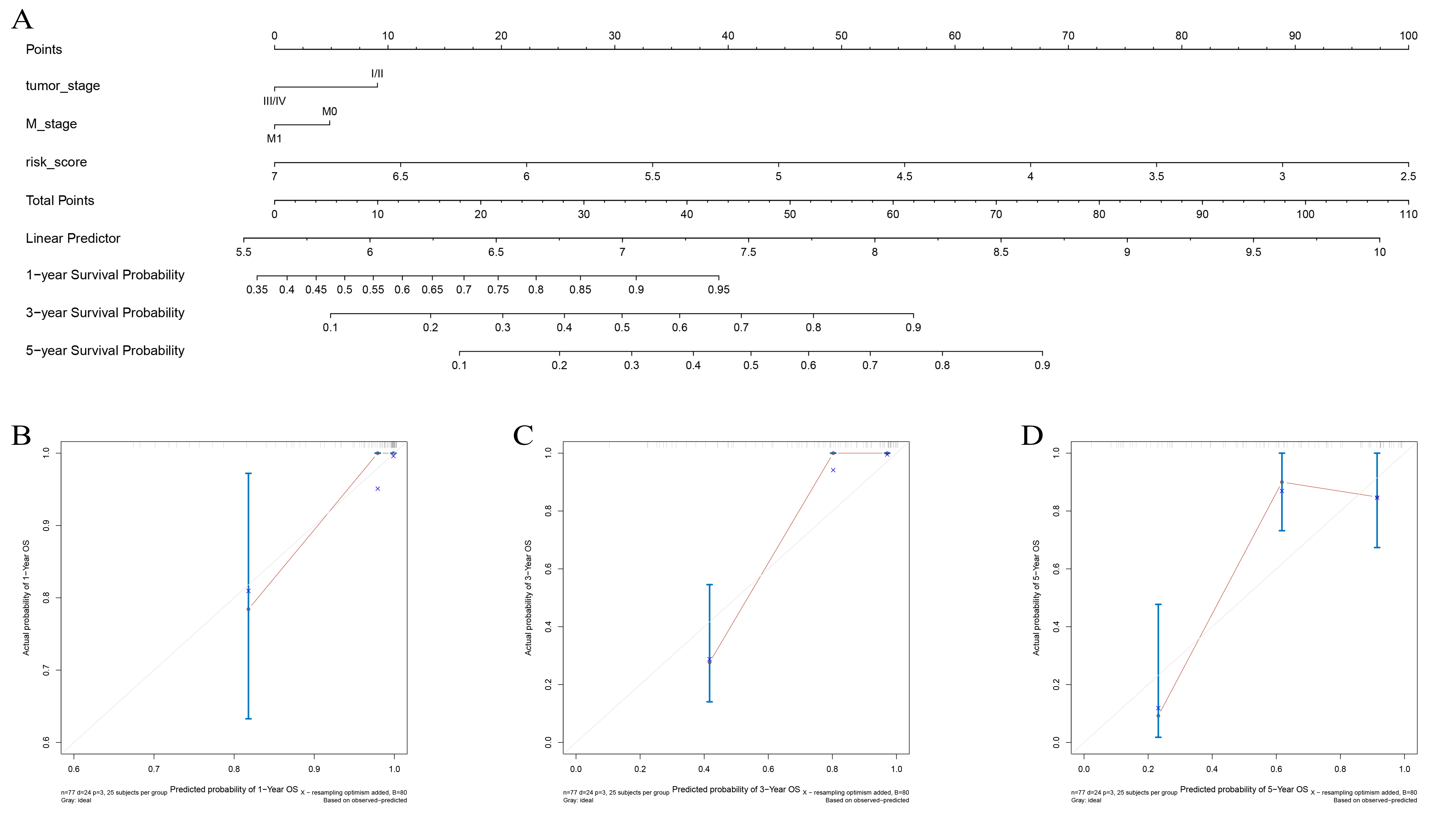

Supplement: Supplementary Figure 8 — Distribution of risk scores of Ridge (A), ELASTIC-NET with α varying from 0.1 to 0.9 (E–J), and lasso model (K) of ACC patients based on TCGA-ACC data. The number of survivors and non-survivors with different risk scores based on TCGA-ACC data; red represents the number of non-survivors, and blue represents the number of survivors. [file Image_8.TIF]

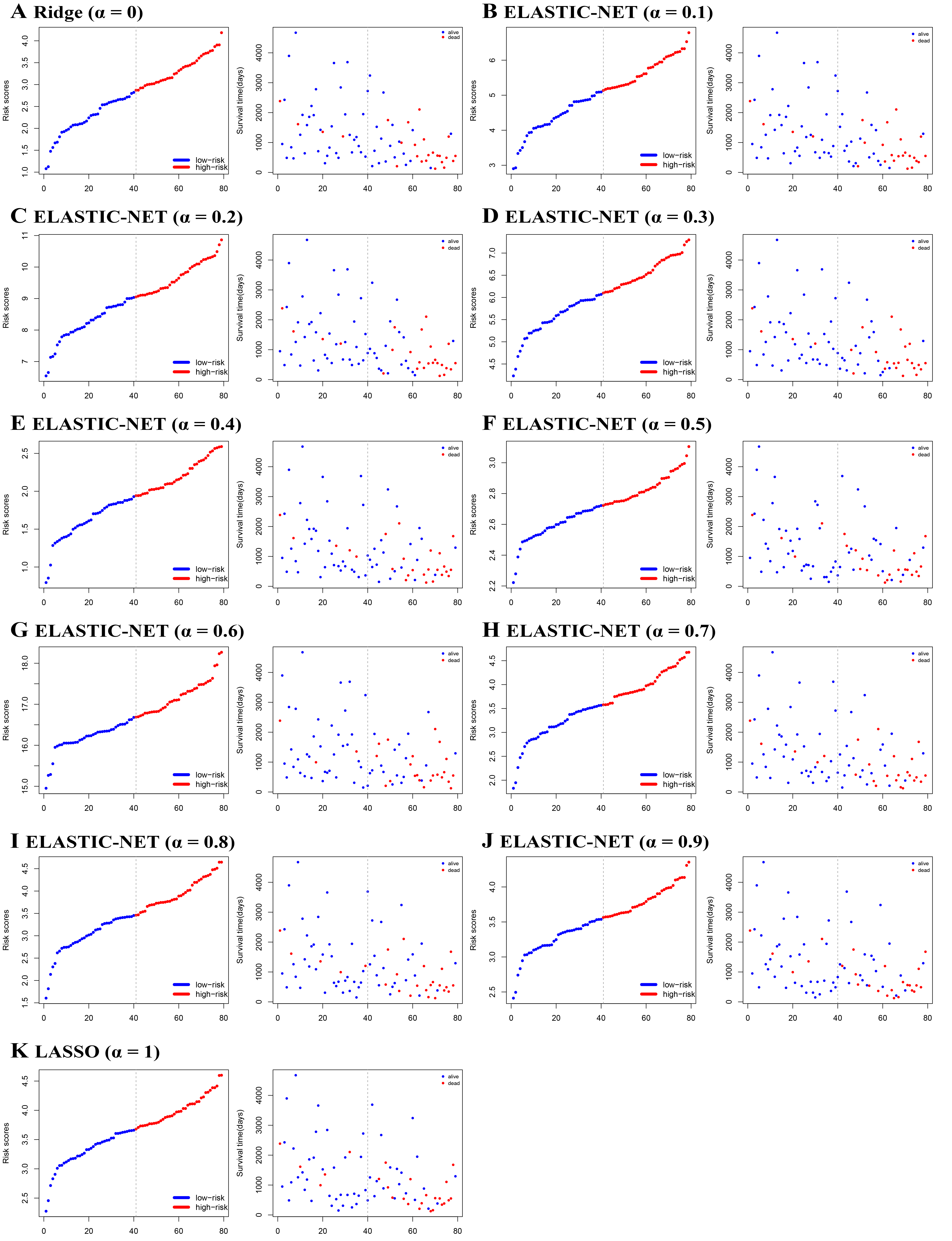

Supplement: Supplementary Figure 9 — (A) The nomogram constructed with model 1 for predicting proportion of patients with 1-, 3- or 5-year OS. The calibration plots for predicting 1- (B), 3- (C) or 5- (D) year OS. OS: overall survival. [file Image_9.TIF]

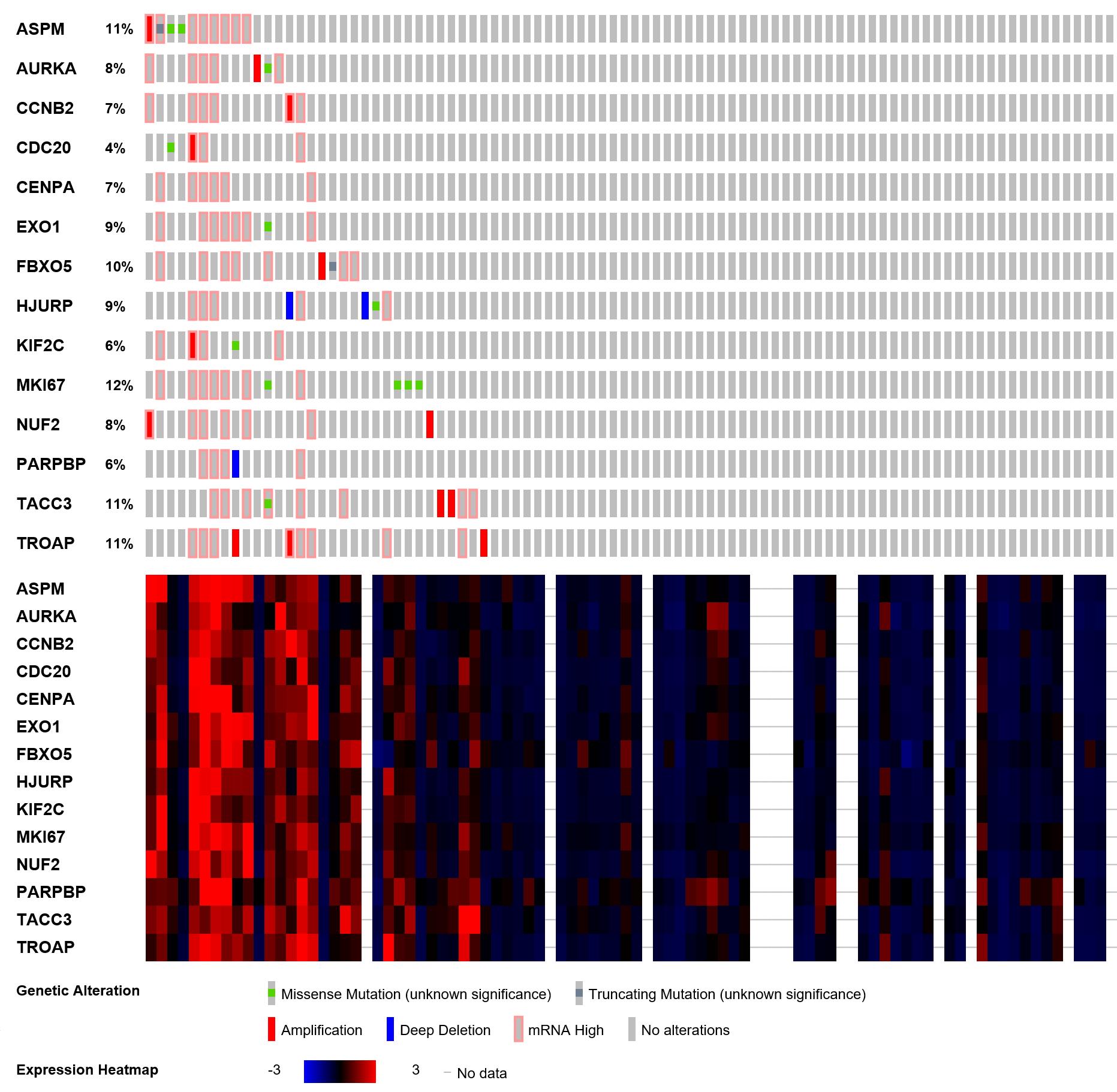

Supplement: Supplementary Figure 10 — Genetic alterations associated with genes in model 2 and expression heatmap of genes in model 2 based on the data from TCGA. [file Image_10.TIF]

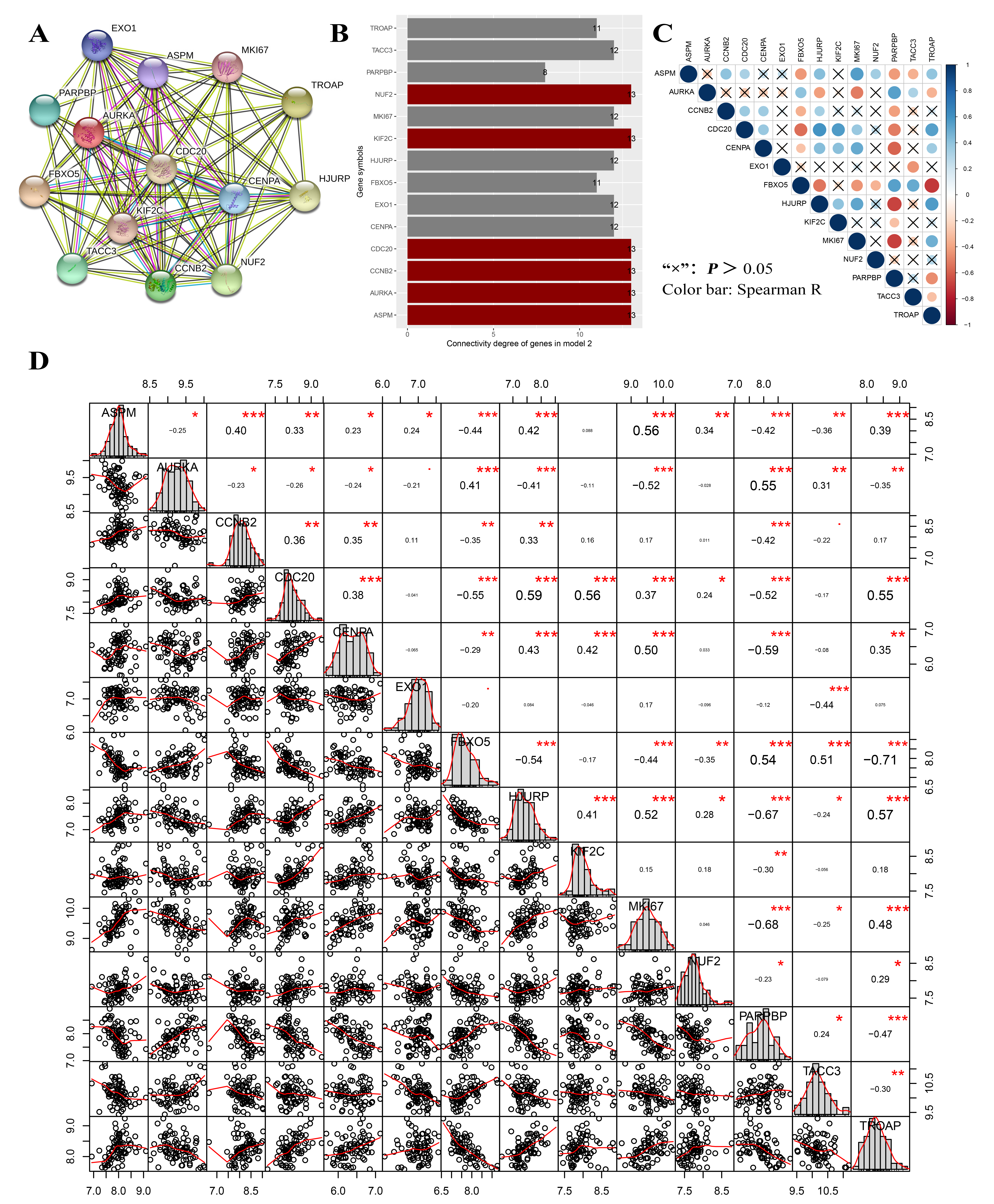

Supplement: Supplementary Figure 11 — Interaction among genes in model 2 and spearman correlation analysis of the genes in model 2. (A) Protein-protein interaction (PPI) network of genes in model 2. (B) Degree of connectivity of genes in model 2 according to the PPI network. (C) Spearman correlation analysis of the genes in model 2. (D) A visual summary for the spearman correlation analysis (scatter plot, bar plot of expression levels of genes in model 2, and spearman correlation coefficients). [file Image_11.TIF]
